# Supplementary material for: Motivations and willingness to provide care from a geographical distance, and the impact of distance care on caregivers’ mental and physical health: a mixed-method systematic review protocol
Source: BMJ Open. 2021 Jul 7;11(7):e045660. doi: 10.1136/bmjopen-2020-045660 (PMC8264892; doi:10.1136/bmjopen-2020-045660)
Supplement: Supplementary data [file bmjopen-2020-045660supp002.pdf]

Motivations and willingness to provide care from a geographic distance, and the impact of distance care on caregivers' mental and physical health: A mixed-method systematic review protocol

Supplementary File 2: Search Strategy – PubMed

#	Search Terms
**#1**	Distance caregiving [Title/Abstract] OR<br><br>Distance caregiver* [Title/Abstract] OR<br><br>Distant caregiving [Title/Abstract] OR<br><br>Distant caregiver* [Title/Abstract] OR<br><br>Distance carer* [Title/Abstract] OR<br><br>Distant carer* [Title/Abstract] OR<br><br>Spatial proximity + care [Title/Abstract] OR<br><br>Care from afar [Title/Abstract]] OR<br><br>Caregiving from afar [Title/Abstract] OR<br><br>Caregiver* from afar [Title/Abstract] OR<br><br>Proximate caregiver* [Title/Abstract] OR<br><br>Proximate caregiving [Title/Abstract] OR<br><br>Proximate carer* [Title/Abstract] OR<br><br>Out of town caregiver* [Title/Abstract] OR<br><br>Immigrant caregiver* [Title/Abstract] OR<br><br>Transnational caregiver* [Title/Abstract] OR<br><br>Transnational caregiving [Title/Abstract]

**#2**	motiv* [Title/Abstract] OR<br><br>motivation* to care [Title/Abstract] OR<br><br>motivation* to provide care [Title/Abstract] OR<br><br>oblig* [Title/Abstract] OR<br><br>filial [Title/Abstract] OR<br><br>duty [Title/Abstract] OR<br><br>guilt* [Title/Abstract] OR<br><br>reciproc* [Title/Abstract] OR<br><br>love [Title/Abstract] OR<br><br>willing* [Title/Abstract] OR<br><br>willingness to care [Title/Abstract] OR<br><br>willingness to provide care [Title/Abstract] OR<br><br>love [MeSH Terms]
**#3**	depress* [Title/Abstract] OR<br><br>distress [Title/Abstract] OR<br><br>stress [Title/Abstract] OR<br><br>mood [Title/Abstract] OR<br><br>anxiety [Title/Abstract] OR<br><br>well being [[Title/Abstract] OR<br><br>mental health [Title/Abstract] OR<br><br>caregiver burden [Title/Abstract] OR<br><br>burden [Title/Abstract] OR<br><br>physical symptoms [[Title/Abstract] OR<br><br>depression [MeSH terms] OR<br><br>anxiety [MeSH terms] OR<br><br>anxiety disorders [MeSH] OR<br><br>mood disorders [MeSH terms] OR

	mental health [MeSH terms] OR<br><br>health [MeSH terms]
**#4**	OR #2 / #3
**#5**	#1 AND #4

Note. * = truncation symbol to identify variations and plurals of words

Motivations and willingness to provide care from a geographic distance, and the impact of distance care on caregivers' mental and physical health: A mixed-method systematic review protocol

Supplementary File 3: Data extraction form (Adapted from Lizarondo *et al.*) [69]

**Study Identification Features**	
**Reviewer**	
**Article Author(s)**	
**Study Title**	
**Date of Publication**	
**Publication Type**	• Research Article<br>- Journal Name:<br>- Record Number:<br>• PhD Thesis/Dissertation<br>• Conference Paper<br>• Other Please Specify: ________________
**Country of Study**	
**Research Aims and Objectives**	
**Methodology**	
**Type of Study (Quantitative, Qualitative or Mixed-method study)**	
**Methodology/Study Design (e.g. Cross-sectional study, Phenomenology)**	
**Method of Analysis (e.g. Thematic analysis, Hierarchical regression analyses)**	
**Method(s) of Data Collection (e.g. Online survey, Interviews, Focus groups)**	
**Inclusion/Exclusion Criteria**	
**Final Sample Size**	
**Recruitment Rate**	
**Drop-out Rate**	
**Time Points (If applicable)**	

**Drop-out Rate for Follow-up Points (If applicable)**		
**Participants' Characteristics**		
**Age**		***$\bar{x}$ (SD)***
**Gender**		***%N***
**Ethnicity**		***%N***
**Care Recipient's Diagnosis**		
**Relationship to Care Recipient**		
**Time Caring**		
**Other Care Experience**		
**Employment Status**		
**Phenomena of Interest**		
**Concepts (C1) - Motivations to Care and/or Willingness to Care**	• Motivations to Care from a Geographic Distance<br>• Willingness to Care from a Geographic Distance<br>• Willingness to Perform Specific Care Tasks (e.g. emotional, nursing or instrumental care tasks)	
**Concepts (C2) – Mental and Physical Health Outcomes**	• Depression<br>• Anxiety<br>• Emotional Distress<br>• Caregiver Burden<br>• Perceived Physical Health<br>• Physical Symptoms	
**Context-related Information**		
**Geographic Distance from the Care Recipient (e.g. assessed using miles or travel time)**		
**Quantitative Findings**		
**Measures**		
**Key Findings**		
**Qualitative Findings**		
**Identified Themes or Subthemes**		
**Author's Conclusions**		
**Reviewer's Comments**		
