# Supplementary material for: Motivations and willingness to provide care from a geographical distance, and the impact of distance care on caregivers’ mental and physical health: a mixed-method systematic review protocol
Source: BMJ Open. 2021 Jul 7;11(7):e045660. doi: 10.1136/bmjopen-2020-045660 (PMC8264892; doi:10.1136/bmjopen-2020-045660)
Supplement: Author's manuscript [file bmjopen-2020-045660.draft_revisions.pdf]

BMJ Open

BMJ Open is committed to open peer review. As part of this commitment we make the peer review history of every article we publish publicly available.

When an article is published we post the peer reviewers' comments and the authors' responses online. We also post the versions of the paper that were used during peer review. These are the versions that the peer review comments apply to.

The versions of the paper that follow are the versions that were submitted during the peer review process. They are not the versions of record or the final published versions. They should not be cited or distributed as the published version of this manuscript.

BMJ Open is an open access journal and the full, final, typeset and author-corrected version of record of the manuscript is available on our site with no access controls, subscription charges or pay-per-view fees (http://bmjopen.bmj.com).

If you have any questions on BMJ Open's open peer review process please email info.bmjopen@bmj.com

BMJ Open

Motivations and willingness to provide care from a geographic distance, and the impact of distance care on caregivers' mental and physical health: A mixed-method systematic review protocol

Journal:	*BMJ Open*
Manuscript ID	bmjopen-2020-045660
Article Type:	Protocol
Date Submitted by the Author:	07-Oct-2020

Complete List of Authors:	Bei, Eva; Bar-Ilan University, Department of Psychology<br>Zarzycki, Mikołaj; Bangor University, School of Psychology<br>Morrison, Valerie; Bangor University, School of Psychology<br>Vilchinsky, Noa; Bar-Ilan University, Department of Psychology

Keywords:	PRIMARY CARE, MENTAL HEALTH, Depression & mood disorders < PSYCHIATRY, Anxiety disorders < PSYCHIATRY

SCHOLARONE™
Manuscripts

I, the Submitting Author has the right to grant and does grant on behalf of all authors of the Work (as defined in the below author licence), an exclusive licence and/or a non-exclusive licence for contributions from authors who are: i) UK Crown employees; ii) where BMJ has agreed a CC-BY licence shall apply, and/or iii) in accordance with the terms applicable for US Federal Government officers or employees acting as part of their official duties; on a worldwide, perpetual, irrevocable, royalty-free basis to BMJ Publishing Group Ltd ("BMJ") its licensees and where the relevant Journal is co-owned by BMJ to the co-owners of the Journal, to publish the Work in this journal and any other BMJ products and to exploit all rights, as set out in our licence.

The Submitting Author accepts and understands that any supply made under these terms is made by BMJ to the Submitting Author unless you are acting as an employee on behalf of your employer or a postgraduate student of an affiliated institution which is paying any applicable article publishing charge ("APC") for Open Access articles. Where the Submitting Author wishes to make the Work available on an Open Access basis (and intends to pay the relevant APC), the terms of reuse of such Open Access shall be governed by a Creative Commons licence – details of these licences and which Creative Commons licence will apply to this Work are set out in our licence referred to above.

Other than as permitted in any relevant BMJ Author's Self Archiving Policies, I confirm this Work has not been accepted for publication elsewhere, is not being considered for publication elsewhere and does not duplicate material already published. I confirm all authors consent to publication of this Work and authorise the granting of this licence.

For peer review only

TITLE

Motivations and willingness to provide care from a geographic distance, and the impact of distance care on caregivers' mental and physical health: A mixed-method systematic review protocol

AUTHORS

Eva Bei (corresponding author), Department of Psychology, Faculty of Social Sciences, Bar-Ilan University, Ramat Gan, 5290002, Israel; ORCID ID: 0000-0002-3093-0829; Email: eva.bei@biu.ac.il; Telephone: +972 0559455802

Mikołaj Zarzycki, School of Psychology, Bangor University, Bangor, Gwynedd, UK; ORCID ID: 0000-0002-8357-563X; Email: m.zarzycki@bangor.ac.uk

Val Morrison, School of Psychology, Bangor University, Bangor, Gwynedd, UK; ORCID ID: 0000-0002-4308-8976; Email: v.morrison@bangor.ac.uk

Noa Vilchinsky, Department of Psychology, Faculty of Social Sciences, Bar-Ilan University, Ramat Gan, 5290002, Israel; ORCID ID: 0000-0003-4965-4745; Email: noa.vilchinsky@biu.ac.il

WORD COUNT: 3659

For peer review only - http://bmjopen.bmj.com/site/about/guidelines.xhtml

ABSTRACT

Introduction

Distance Caregivers (DCGs) are a growing population with substantial contribution to informal care. While a reasonable amount is known on the determinants of motives and willingness to provide local informal care, and the local caregiver outcomes, reports for the distance caregiving population are lacking. An evidence synthesis of what motivates and makes DCGs willing to care from a distance and the impact of that care on their mental and physical health, would highlight any gaps or consensus in knowledge. This would guide the research needed towards the development of tailored interventions, in order to support DCGs and promote the sustainability of distance care.

Methods and analysis

This protocol adheres to Preferred Items for Reporting of Systematic Reviews and Meta-Analyses Protocols (PRISMA) guidelines and the Joanna Briggs Institute (JBI) Methodology for mixed-method reviews. A comprehensive search strategy will be conducted in four electronic databases (CINAHL, MEDLINE, PubMed and PsycINFO). Grey literature will be also assessed to minimise publication bias. Two independent reviewers will assess each study for inclusion and any discrepancies will be resolved with the consultation of a third reviewer. Eligible studies for inclusion will be English language studies exploring the motives and willingness to care for a care recipient with a chronic disease, disability or frailty from a geographic distance; or studies focusing on the mental and physical health outcomes of DCGs. Qualitative and quantitative data will be integrated in a single qualitative synthesis following the JBI convergent integrated approach. Study quality will be assessed using the Mixed Methods Appraisal Tool (MMAT) version 2018.

Ethics and dissemination

Ethical approval is not required for this study as no primary data will be collected. Findings will be disseminated through peer-reviewed publication and presentations at academic conferences and lay summaries for various stakeholders.

PROSPERO registration number CRD42020156350.

STRENGTHS AND LIMITATIONS OF THIS STUDY

- To the best of our knowledge no previous study has systematically explored the motivations and willingness to provide care among DCGs, and the impact of distance care on caregivers' mental and physical health.
- A mixed-method review will set the groundwork and inform research needed to guide the development of tailored interventions to support DCGs and promote the sustainability of distance care.
- A rigorous, systematic approach will be applied to searching, screening, extracting and analysing evidence in four different academic databases and in grey literature.
- Anticipated potential limitations include high degree of heterogeneity across studies as there is no consensus on the definition of distance caregiving which makes it difficult to compare studies.
- The review will be restricted to studies published only in English, which may cause language bias.

KEY WORDS

Distance caregiving, geographic distance, motivations to care, willingness to care, mental health, physical health

INTRODUCTION

With the ageing of the population worldwide, numbers of older and care-dependent adults are rising [1-5]. Informal caregivers who provide unpaid care to a family member or friend with long-term care needs, are an essential pillar for sustaining healthcare systems worldwide and maintaining outpatient care [6-8]. Traditional patterns of informal care usually involved family or friends co-residing or living close to the care recipient [9]. However, as the demand for caregivers continues to grow, social changes such as urbanisation, gendered roles within society, increased labor market mobility and globalisation have affected the traditional ways of providing care [9-11]. Distance care has emerged in those cases where adult caregivers are not staying with their care recipient but making efforts for providing informal care from a geographic distance [11-13].

It is estimated that 15–20% of all informal care is provided by Distance Caregivers (DCGs) [12]. One of the problems in this field of research is that there is no consensus on how to best define distance caregiving. Most research to date has used mileage [12] or travel time

For peer review only - http://bmjopen.bmj.com/site/about/guidelines.xhtml

categories [14,15] to measure the geographic distance from the care recipient and define DCGs. Overall, travel time and space along with a number of socioeconomic factors which are confounded with distance, such as travel costs and access to transportation, will influence distance caregiving - in its existence as well as in its extent [16].

In their previous review, Cagle and Munn [17] suggested that the definition of distance care as articulated by Parker *et al.* [18], is the most comprehensive and compelling. Indeed, the definition appears to be appropriate as it operationalises geographic distance without focusing exclusively on distance and travel time but also on a number of other socioeconomic factors that determine geographic distance and affect distance care provision. According to Parker *et al.*, a DCG could be defined as: "Anyone (1) who provides informal, unpaid care to a person experiencing some degree of physical, mental, emotional, or economic impairment that limits independence and necessitates assistance; and (2) who experiences caregiving complications because of geographic distances from the recipient, as determined by distance, travel time, travel cost, personal mobility problems, limited transportation, and other related factors that affect the caregiver's access to the care recipient" [18].

Demographic analyses show that most DCGs are adult children providing assistance to a family member, usually an aged parent, parent in-law or step-parent with a chronic health condition, disability or other long-lasting healthcare needs [17,19]. DCGs engage in many activities to ensure that the needs of their care recipient are met, such as providing social and emotional support, managing financial affairs, monitoring and coordinating care and healthcare services [12, 20-22]. Although most DCGs are unable to provide hands-on care on a daily basis as local caregivers, they are significantly involved in performing practical and nursing tasks when visiting their loved one. In fact, approximately 75% provide assistance with Instrumental Activities of Daily Living (IADLs) such as household tasks, cooking, grocery shopping, medical care and transportation [12]. Also, 40% report assisting with Activities of Daily Living (ADLs) such as bathing, dressing, feeding and toileting [12].

Motivations and willingness to provide informal care

In recent years, a growing body of literature has explored caregivers' motivations and willingness to carry out a caregiving role [23-25]. Research on local caregiving has identified multiple and often inter-related determinants of caregivers' motives and willingness to care for a loved one [23]. Informal care is likely to be provided out of love and affection, reflecting a long-lasting family or friend relationship between the caregiver and the care

For peer review only - http://bmjopen.bmj.com/site/about/guidelines.xhtml

recipient [26,27]. Reciprocity is also a frequently cited motive for informal care provision. Adult children or children in-law often reciprocate the care and love received in the past from the care recipient, by undertaking the caregiving role [28-32]. In addition, caregiving is frequently described as a family duty, filial obligation or responsibility. Adult children are often motivated to care as a means of fulfilling their care responsibilities, in socio-cultural contexts where caregiving is viewed as an unspoken family value, a moral obligation [28, 33-37]. Beside these factors, availability of other caregivers [30,34] and resources for formal care [38] may also have an effect on the provision of informal care.

Baldassar and colleagues [21] demonstrated that DCGs may describe some similar motives to care to those of local caregivers. Despite the unique challenges and complications caused by physical separation, DCGs felt that they were greatly indebted to their parents for raising them and it was now their turn to reciprocate the care received and pay them back even from a distance [21]. They also reported a strong sense of obligation to care based on moral and societal expectations regarding caretaking duties [21]. However, DCGs may also report motives to care unique to the distance caregiving situation, for example, in a recent study [39], DCGs expressed feelings of guilt about not being physically present to fulfil their duty to care. Although physical distance limited DCGs from providing care on a regular basis, it also worked as a strong motivator, increasing their willingness and efforts for providing adequate care from afar [39].

Previous findings have highlighted the significance of motivations and willingness to take on the caregiving role. Camden and colleagues [40] found that those who were unwilling to provide care, reported higher abusive behaviors towards the care recipient while their loved one was more likely to be admitted to a care home the following year. Another qualitative study showed that caregivers who provided care out of societal expectations had less control over the caregiving challenges [41]. Additionally, Romero-Moreno *et al.* [24] reported that extrinsic motives for caring (i.e. filial obligation) are associated with poorer caregivers' mental health. Similarly, in their systematic review, Quinn *et al*. [25] found that caregivers motives to provide informal care can have implications on their psychological well-being.

Mental and physical health outcomes of informal caregiving

Studies of broader caregiving samples suggest that caregivers can experience both positive and negative outcomes as a result of their caretaking responsibilities [42-44]. The positive aspects of caregiving may include an enhanced relationship with the care recipient, feelings

For peer review only - http://bmjopen.bmj.com/site/about/guidelines.xhtml

For peer review only

of personal growth, gains and satisfaction, and an overall rewarding and meaningful experience [8,42,45-47]. However, caregivers may also experience negative outcomes related to the caregiving role, such as poor mental and physical health [47-48]. Numerous studies have identified high levels of caregiver burden across a wide range of chronic diseases, as a multidimensional response to the various stressors associated with caring [48-50]. In terms of psychological morbidity, high levels of emotional distress and increased symptoms of anxiety and depression have been reported [51-53] with, for example, a meta-analytical review reporting a prevalence of anxiety and depressive symptomatology among caregivers of stroke survivors as above 20% and 40%, respectively [52]. In addition, many caregivers experience poor physical health, related to their role [54-56]. A meta-analysis of 84 studies conducted across a range of health conditions revealed that caregivers reported statistically significant lower levels of well-being and physical health than non-caregivers [55], with some specific findings pointing to caregivers showing poorer immune response [57] and exhibiting greater cardiovascular reactivity [56] than non-caregivers.

Geographic distance creates additional burdens for what is already often a stressful care work [17]. Koerin and colleagues [58] found that almost 80% of DCGs reported emotional distress related to feelings of inadequacy on how to assess the needs of their loved one from afar and uncertainty regarding the progression of their illness. Additionally, in their recent study, Li *et al.* [14] found that adult child primary caregivers who live more than 30 minutes away from their frail older parents had higher levels of depression than co-residing caregivers. Findings also showed that although co-residing caregivers were more distressed by objective caregiver burden, DCGs were more distressed by subjective caregiver burden which includes perceptions and attitudes related to caregiving [14].

The negative mental and physical health outcomes of caregiving can threaten caregivers' commitment to the welfare of the care recipient and reduce the quality of care provision [59,60]. Additionally, poor caregiver outcomes are associated with adverse health outcomes for care recipients, including higher levels of emotional distress, elder abuse, mortality and hospitalization [61-63]. Increased morbidity of caregivers can also lead to high individual and societal burdens and affect the sustainability of informal care [63,64].

For peer review only - http://bmjopen.bmj.com/site/about/guidelines.xhtml

Aim of the review

DCGs are a growing population who make a substantial contribution to informal care. To-date, to the best of our knowledge, no review has systematically evaluated, and quality appraised the current evidence on motivations and willingness to care from a distance, and the impact of distance care on caregivers' mental and physical health outcomes. Most systematic reviews have currently focused on local caregivers [23,24,65], excluding the distance caregiving population. In addition, past reviews of distance caregiving have been limited to evaluating evidence on the phenomenon of distance care in general, focusing on the definition and sociodemographic characteristics of DCGs and describing only briefly the benefits and costs of such a role [17,66]. Other reviews have exclusively focused on distance caregiving for patients with advanced cancer without exploring distance care on other health conditions with different care needs or demands [13]; or on the availability of technology-based and eHealth interventions to support DCGs [67].

However, as previously stated, DCGs may report unique experiences and motives to care, associated with the geographic and spatial distance from the care recipient [21,39]. Experiencing the added challenges and complications of caring from afar, DCGs may also be at higher risk of poor mental and physical health outcomes [14,17,58]. A systematic review would synthesise evidence from studies on what motivates DCGs and makes them willing to provide care from a distance, and what is the impact of that care on their mental and physical health. Further understanding of these issues, would provide evidence for the development of geographically sensible and tailored interventions in order to promote the sustainability of distance care and support those caring from afar. Therefore, this review aims to:

1) Synthesise and critique the evidence on the determinants of motivations and willingness to care from a geographic distance.
2) Synthesise and critique the evidence on the impact of distance caregiving on caregivers' mental and physical health.

For peer review only - http://bmjopen.bmj.com/site/about/guidelines.xhtml

METHODS AND ANALYSIS

This protocol is guided by the Preferred Items for Reporting of Systematic Reviews and Meta-Analyses Protocols (PRISMA) checklist [68] (Online supplementary appendix 1) and the Joanna Briggs Institute Methodology for Mixed-Methods Systematic Reviews [69].

Study registration

Based on the PRISMA guidelines [68], the protocol for this systematic review was registered on the international database of prospectively registered systematic reviews in health and social care, PROSPERO (registration number: CRD42020156350). Any important protocol amendments will be recorded in PROSPERO and published with the results of the review.

Eligibility criteria

Types of studies

Eligible studies for inclusion will be English language peer-reviewed studies or unpublished studies such as doctoral theses in order to reduce publication bias. These will include quantitative studies (analytical observational and descriptive observational studies), studies that focus on qualitative data and mixed-method study designs. The inclusion of different types of research will provide more informative findings for each of the two review objectives and increase the ability of those findings to inform future research, policy and practice.

Participants

The population of interest are adult DCGs (aged 18 and above) of adult family members or friends with healthcare needs. The comprehensive definition of distance care by Parker *et al.* [18] was used and adapted for the purposes of this review, and in order to maximize our access to empirical studies that conceptualised geographic distance from the care recipient in different ways. Thus, as a DCG could be defined:

(1) Anyone who provides informal, unpaid care to a relative or friend with a chronic illness, disability or frailty that limits independence and necessitates assistance;

AND

(2) who experiences caregiving complications because of geographic distances from the care recipient, as determined by distance, travel time, travel cost, personal mobility problems, limited transportation, or other related factors that affect the caregiver's access to the care recipient.

For peer review only - http://bmjopen.bmj.com/site/about/guidelines.xhtml

Care recipient's health conditions eligible for this review will include any chronic illness and disability (e.g. cancer, dementia, cardiovascular disease, stroke etc.) or simply frailty. Studies exclusively focusing on young DCGs (under 18 years old) or DCGs of children and adolescents will be excluded, as young caregiving [70] and care receiving [71] experiences are associated with additional needs and burdens.

Outcomes

Primary outcomes

The primary outcomes of interest are caregivers' motives and willingness to provide care from a geographic distance. These may include qualitative and/or quantitative data. Qualitative findings describing various determinants of motives and willingness to care from a distance such as for example family values, love and affection, reciprocity, guilty feelings, filial obligation and specific illness characteristics will be reported. In addition, qualitative studies exploring willingness to perform a variety of caregiving tasks from afar or when visiting the care recipient, such as emotional, nursing and instrumental care tasks, will be considered. For quantitative findings, studies must report on motives and willingness to care from a distance, assessed using a validated self-report instrument (e.g. Motivations in Elder Care Scale [72], Willingness to Care Scale [73]) or a self-report instrument developed for the purposes of the included study. Given that many studies explored the determinants of motives and willingness to provide local care using qualitative methodology, it is envisaged that our outcomes will be also primarily explored through qualitative data.

Secondary outcomes

Secondary outcomes of interest are those studies assessing the impact of distance care provision on caregivers' mental and physical health outcomes, specifically depression, anxiety, emotional distress, caregiver burden, perceived physical health or physical symptoms. Eligible studies for inclusion will be quantitative studies that assessed distance caregiver outcomes using a validated self-report outcome instrument or an instrument developed for the purposes of the included study. Examples of validated psychrometric instruments for the secondary outcomes of interest include the Centre for Epidemiologic Studies-Depression Scale (CES-D) [74], the Hospital Anxiety and Depression Scale (HADS) [75], the Profile of Mood States (POMS) [76], the Perceived Stress Scale [77], the Zarit Burden Interview [78] or the Cohen-Hoberman Inventory of Physical Symptoms (CHIPS)

For peer review only - http://bmjopen.bmj.com/site/about/guidelines.xhtml

[79]. Qualitative findings exploring manifestations and perceptions of emotional distress, anxiety, depression, burden and physical health will be also considered.

Search strategy

A detailed search strategy without limits on publication date, will be conducted on four relevant electronic databases (CINAHL, MEDLINE, PubMed and PsycINFO), to comprehensively search for studies on distance caregiving. Grey literature, including the OpenGrey (http://www.opengrey.eu/) and Electronic Theses Online Service (ETHOS; https://ethos.bl.uk/) databases, will be also searched to maximize our access to potentially relevant studies and reduce publication bias. In addition, the reference lists of all studies included will be screened to identify additional citations of interest.

The search strategy was developed using key terms related to the distance caregiving population and the primary and secondary outcomes of interest. The search was also informed by past reviews on distance care [13,17]. A variation of controlled vocabulary, relevant Medical Subject Headings (MeSH) when possible and free-text terms contained in the title/abstracts of publications will be applied. An example of the search strategy string terms used for PubMed database is presented (Online supplementary appendix 2). Search strategy string will be then adapted to the remaining three electronic databases. Per database these terms will be mapped to Subject Headings. Included studies will be restricted to those written in English for ease of interpretation.

Study Selection

Following the search, all identified citations will be imported into Zotero and duplicates removed. Selection process of the relevant studies will be conducted by two independent reviewers (EB and MZ). One reviewer (EB) will screen the titles of each study based on the inclusion criteria and when these are deemed relevant, she will go through abstracts. Once narrowed down by abstracts, a full-text review process will be completed in duplicate by two reviewers (EB and MZ) for studies that met the eligibility criteria at screening and for studies with unclear relevance. A third independent author (NV) will be consulted to resolve any discrepancies that arose between the two reviewers during the screening process. Additional records identified from searches of grey literature and reference lists will be also assessed in detail against the eligibility criteria by the principal researcher (EB). Original authors of studies identified will be contacted if the full-text paper was not available or the relevance of a paper was unclear. The PRISMA flow diagram will outline the process of study selection

For peer review only - http://bmjopen.bmj.com/site/about/guidelines.xhtml

(Figure 1) [68]. Reasons for exclusion of full-text studies that do not meet the eligibility criteria will be also recorded in the flow diagram.

Figure 1

Assessment of methodological quality

To assess the methodological quality of the included studies, the Mixed Methods Appraisal Tool (MMAT) version 2018 will be used, a quality appraisal instrument for mixed-method reviews [80]. The MMAT has been content validated and piloted across all methodologies [80]. The instrument consists of two screening questions for all study designs and five sections of specific questions regarding study type. The quality of each study will be assessed as low (0-1/5 criteria met), medium (2-3/5 criteria met) or high (4-5/5 criteria met), using the total number of MMAT criteria that were met. Quality appraisal will be completed in duplicate by two independent reviewers (EB and MZ). Any discrepancies will be resolved with the consultation of a third reviewer (VM). The strength of the body of evidence will be discussed and narratively incorporated into the synthesis. No study will be excluded on the basis of their methodological quality, as they could still offer valuable insight. Yet, findings of the studies assessed as low will be interpreted with caution.

Data Extraction

Data extraction process will be carried out using the JBI data extraction tool [69] as adapted and modified for the purposes of this review (Online supplementary appendix 3). Data will be extracted for each study by one reviewer (EB) and independently extracted by the second reviewer (MZ) for accuracy, employing a double-coding on portion of 80% of the included studies. Any discrepancies will be resolved with the consultation of a third reviewer (NV). Data extracted from the included studies will comprise research aims and objectives, study methods, population characteristics, phenomena of interest and context-related information, main study findings and relevant outcomes. Extracted information will vary across different study designs.

Data synthesis

Data synthesis will involve a convergent integrated approach, as per JBI methodology for questions that can be addressed by both quantitative and qualitative research designs (Figure

For peer review only - http://bmjopen.bmj.com/site/about/guidelines.xhtml

2) [69]. Extracted data from quantitative findings will be first converted into “qualitised data”. This will involve a narrative interpretation of the quantitative results and the transformation of numerical data into textual descriptions. The “qualitised” data will be then assembled and pooled with the results of qualitative studies. A detailed examination of the pooled data will be finally undertaken to identify categories based on similarity and produce a set of integrated findings for each of the two review objectives [69].

Figure 2

CONCLUSION

To the best of our knowledge, this is the first proposed review to systematically examine DCGs’ motivations and willingness to care, and the impact of distance care on caregivers’ mental and physical health. Given that distance caregiving is a distinct phenomenon which differs substantially from on-site care [13], findings of this review will inform researchers, healthcare practitioners and policy makers on the specific determinants of motives and willingness to care from afar and the unique needs and burdens of this subpopulation of carers. In addition, results will provide evidence for the development and implementation of geographically sensible and tailored interventions in order to address the complex needs of DCGs and promote the sustainability of distance care.

PATIENT AND PUBLIC INVOLVEMENT

Patients and members of the public were not involved in the design and development of this protocol.

ETHICS AND DISSEMINATION

Ethical approval and consent to participate are not required for the proposed systematic review as no primary data will be collected. Findings will be disseminated through publication in an international peer reviewed journal and presented at local and international scientific conferences on informal care and health psychology. A lay summary of the review will be written for healthcare organisations and non-scientific audiences such as caregivers and their care recipients and disseminated through mass emails, social media, blogs and appropriate webpages.

For peer review only - http://bmjopen.bmj.com/site/about/guidelines.xhtml

AUTHORS' CONTRIBUTIONS

EB contributed to the conception and design of the study and wrote the manuscript. NV contributed to the conception and design of the study and critically revised the manuscript draft. MZ and VM critically revised the study design and the manuscript draft. All authors approved the final manuscript.

FUNDING

This work is funded by the European Union's Horizon 2020 research and innovation programme under the Marie-Sklodowska Curie grant agreement no 814072.

DISCLAIMER

The funder had no role in this protocol's development.

COMPETING INTERESTS

None declared.

PATIENT CONSENT FOR PUBLICATION

Not required.

ABBREVIATIONS

CINAHL: Cumulative Index to Nursing and Allied Health Literature

PRISMA-P: Preferred Reporting Items for Systematic Reviews and Meta-Analysis Protocols

PROSPERO: International prospective register of systematic reviews

REFERENCES

1. Casal BR, Rivera BC, Currais LN. Alzheimer's disease and the quality of life of the informal caregiver. *Rev Esp Geriatr Gerontol* 2019;54:81-7. doi:10.1016/j.regg.2018.10.008
2. Li J, Han X, Zhang X, *et al*. Spatiotemporal evolution of global population ageing from 1960 to 2017. *BMC public health* 2019; 19:127. doi:10.1186/s12889-019-6465-2
3. Sanai SA, Saini V, Benedict RH, *et al*. Aging and multiple sclerosis. *Mult Scler J* 2016;22:717-25. doi:10.1177/1352458516634871
4. Spasova S, Baeten R, Coster S, *et al*. *Challenges in long-term care in Europe: A study of national policies*. Brussels: European Comission: European Social Policy Network (ESPN), 2018.
5. Verbakel E. How to understand informal caregiving patterns in Europe? The role of formal long-term care provisions and family care norms. *Scand J Public Healt 2018*;46:436-47.
6. Colombo F, Llena-Nozal A, Mercier J, *et al*. Help wanted? providing and paying for long-term care. OECD Publishing 2011. doi:10.1787/9789264097759-en
7. Revenson T, Griva K, Luszczynska, *et* al. Caregiving in the illness context. Hampshire: Springer 2016. doi:10.1057/9781137558985
8. Roth DL, Fredman L, Haley WE. Informal caregiving and its impact on health: A reappraisal from population-based studies. *Gerontologist* 2015;55:309-19. doi: 10.1093/geront/gnu177
9. Baldassar L, Baldock CV, Wilding R. Families caring across borders: Migration, ageing and transnational caregiving. Springer 2006.
10. Croucher K, Gilroy R, Bevan M, *et al*. The mobilities of care in later life: exploring the relationship between caring and mobility in the lives of older people. *Ageing Soc* 2020;1-22. doi:10.1017/S0144686X20000100
11. Kaushik A. Elder Care from a Distance: Emerging Trends and Challenges in the Contemporary India. In: Shankardass M. Ageing Issues and Responses in India. Springer, Singapore 2020:97-113. doi: 10.1007/978-981-15-5187-1_6
12. National Alliance for Caregiving. Miles away: The MetLife study of long-distance caregiving. New York, NY: MetLife Mature Market Institute 2004:1-13.
13. Douglas SL, Mazanec P, Lipson A, *et al*. Distance caregiving a family member with cancer: A review of the literature on distance caregiving and recommendations for future research. *World J Clin Oncol* 2016;7:214. doi:10.5306/wjco.v7.i2.214
14. Li M, Mao W, Chi I, *et al*. Geographical proximity and depressive symptoms among adult child caregivers: social support as a moderator. *Aging Ment Health* 2019;23:205-13. doi: 10.1080/13607863.2017.1399349
15. Roff LL, Martin SS, Jennings LK., *et al*. Long distance parental caregivers' experiences with siblings: A qualitative study. *Qual Soc Work* 2007;6:315–34. doi:10.1177/ 1473325007080404
16. Bei E, Rotem-Mindali O, Vilchinsky N. Providing Care From Afar: A Growing Yet Understudied Phenomenon in the Caregiving Field. *Front Psychol* 2020;11:681. doi: 10.3389/fpsyg.2020.00681
17. Cagle JG, Munn JC. Long-distance caregiving: a systematic review of the literature. *J Gerontol Soc Work* 2012;55:682-707. doi:10.1080/01634372.2012.703763

For peer review only - http://bmjopen.bmj.com/site/about/guidelines.xhtml

18. Parker MW, Church W, Toseland RW. Caregiving at a Distance. In: Berkman B, D'Ambruoso S, eds. Handbook of Social Work in Health and Aging. New York: Oxford University Press 2006: 391-406. doi:10.1093/acprof:oso/9780195173727.003.0033
19. Mazanec P. Distance caregiving a parent with cancer. *Semin Oncol Nurs* 2012;28:271-78. doi: 10.1016/ j.soncn.2012.09.010
20. Harrigan MP, Koerin BB. Long distance caregiving: Personal realities and practice implications. *Reflections (Long Beach)* 2007; 13:5–16.
21. Baldassar L. Transnational families and aged care: The mobility of care and the migrancy of ageing. *J Ethn Migr Stud* 2007;33:275-97. doi:10.1080/13691830601154252
22. Baldock CV. Migrants and their parents: Caregiving from a distance. *J Fam Issues* 2000;21:205-24. doi: 10.1177/019251300021002004
23. Greenwood N, Smith R. Motivations for being informal carers of people living with dementia: a systematic review of qualitative literature. *BMC Geriatr* 2019;19:169. doi:10.1186/s12877-019-1185-0
24. Romero-Moreno R, Márquez-González M, Losada A, *et al*. Motives for caring: relationship to stress and coping dimensions. *Int Psychogeriatr* 2011,23:573. doi:10.1017/S1041610210001821
25. Quinn C, Clare L, Woods RT. The impact of motivations and meanings on the well-being of caregivers of people with dementia: a systematic review. *Int Psychogeriatr* 2010; 22:43–55. doi:10.1017/S1041610209990810
26. Lin MC, Macmillan M, Brown N. A grounded theory longitudinal study of carers' experiences of caring for people with dementia. *Dementia* 2011;11:181–97. doi: 10.1177/1471301211421362
27. McDonnell E, Ryan AA. The experience of sons caring for a parent with dementia. *Dementia*. 2014;13:788–802. doi: 10.1177/1471301213485374
28. Donorfio LK, Kellett K. Filial responsibility and transitions involved: A qualitative exploration of caregiving daughters and frail mothers. *J Adult Dev* 2006;13:158-67. doi:10.1007/s10804-007-9025-4
29. Qadir F, Gulzar W, Haqqani S, *et al*. A pilot study examining the awareness, attitude, and burden of informal caregivers of patients with dementia. *Care Manag J* 2013;14:230–40. doi: 10.1891/1521-0987.14.4.230
30. Quinn C, Clare L, Woods RT. Balancing needs: The role of motivations, meanings and relationship dynamics in the experience of informal caregivers of people with dementia. *Dementia* 2015;14:220-37. doi:10.1177/1471301213495863
31. Santos RL, Sousa MF, Ganem AC, *et al*. Cultural aspects in dementia: differences in the awareness of Brazilian caregivers. *Trends Psychiatry Psychother* 2013; 35:191-7. doi: 10.1590/s2237-60892013000300006
32. van Wezel N, Francke AL, Kayan-Acun E, *et al*. Family care for immigrants with dementia: the perspectives of female family carers living in the Netherlands. *Dementia* 2016;15:69–84. doi: 10.1177/1471301213517703
33. Dunér A. Motives, experiences and strategies of next of kin helping older relatives in the Swedish welfare context: a qualitative study. *Int J Soc Welf* 2010;19:54-62. doi: 10.1111/j.1468-2397.2008.00631.x
34. Parveen S, Morrison V, Robinson CA. Ethnicity, familism and willingness to care: Important influences on caregiver mood? *Aging Ment Health* 2013;17:115-24. doi:10.1080/13607863.2012.717251
35. Parveen S, Morrison V, Robinson CA. Ethnic variations in the caregiver role: A qualitative study. *J Health Psychol* 2011;16:862-72. doi: 10.1177/1359105310392416

36. Kim Y. Korean American family post caregivers on dementia caregiving: a phenomenological inquiry. *J Gerontol Soc Work* 2009;52:600–17. doi: 10.1080/01634370903048352
37. Yoo GJ, Kim BW. Remembering sacrifices: Attitude and beliefs among second-generation Korean Americans regarding family support. *J Cross Cult Gerontol* 2010;25:165-81. doi:10.1007/s10823-010-9116-8
38. Chang YP, Schneider JK, Sessanna L. Decisional conflict among Chinese family caregivers regarding nursing home placement of older adults with dementia. *J Aging Stud* 2017;25:436–44. doi:10.1016/j.jaging.2011.05.001
39. Amin I, Ingman S. Eldercare in the transnational setting: Insights from Bangladeshi transnational families in the United States. *J Cross Cult Gerontol* 2014;29:315-28. doi:10.1007/s10823-014-9236-7
40. Camden A, Livingston G, Cooper C. Reasons why family members become carers and the outcome for the person with dementia: results from the CARD study. *Int Psychogeriatr* 2011;23:1442. doi:10.1017/S1041610211001189
41. Ng HY, Griva K, Lim HA, *et al*. The burden of filial piety: A qualitative study on caregiving motivations amongst family caregivers of patients with cancer in Singapore. *Psychol Health* 2016;31:1293-310. doi:10.1080/08870446.2016.1204450
42. Li Q, Loke AY. The positive aspects of caregiving for cancer patients: a critical review of the literature and directions for future research. *Psycho-Oncol* 2013;22:2399-407. doi:10.1002/pon.3311
43. Lambert SD, Levesque JV, Girgis A. The impact of cancer and chronic conditions on caregivers and family members. In: Koczwara. Cancer and chronic conditions: Addressing the Problem of Multimorbidity in Cancer Patients and Survivors Springer, Singapore 2016:159-202. doi:10.1007/978-981-10-1844-2_6
44. Wang Z, Ma C, Han H, *et al*. Caregiver burden in Alzheimer's disease: Moderation effects of social support and mediation effects of positive aspects of caregiving. *Int J Geriatr Psychiatry* 2018;33:1198-206. doi:10.1002/gps.4910
45. Parveen S, Morrison V. Predicting caregiver gains: A longitudinal study. *Br J Health Psychol* 2012;17:711–23. doi:10.1111/j.2044-8287.2012.02067.x
46. Kim Y, Schulz R, Carver CS. Benefit finding in the cancer caregiving experience. *Psychosom Medicine* 2007;69:283-91. doi:10.1097/PSY.0b013e3180417cf4
47. Schulz R, Sherwood PR. Physical and mental health effects of family caregiving. *J Soc Work Educ* 2008;44:105-113. doi:10.5175/JSWE.2008.773247702
48. Allen AP, Curran EA, Duggan A, *et al.* A systematic review of the psychobiological burden of informal caregiving for patients with dementia: Focus on cognitive and biological markers of chronic stress. *Neurosci Biobehav Rev* 2017;73:123-64. doi:10.1016/j.neubiorev.2016.12.006
49. Chiao CY, Wu HS, Hsiao CY. Caregiver burden for informal caregivers of patients with dementia: a systematic review. *Int Nurs Rev* 2015; 62:340–50. doi:10.1111/inr.12194
50. Faronbi JO, Faronbi GO, Ayamolowo SJ, *et al*. Caring for the seniors with chronic illness: the lived experience of caregivers of older adults. *Arch Gerontol Geriatr* 2019;82:8–14. doi: 10.1016/j.archger.2019.01.013
51. Geng HM, Chuang DM, Yang F, *et al.* Prevalence and determinants of depression in caregivers of cancer patients: A systematic review and meta-analysis. *Medicine* 2018;97:e11863. doi: 10.1097/MD.0000000000011863

For peer review only - http://bmjopen.bmj.com/site/about/guidelines.xhtml

52. Loh AZ, Tan JS., Zhang MW, *et al*. The global prevalence of anxiety and depressive symptoms among caregivers of stroke survivors. *J Am Med Dir Assoc* 2017;18:111-116. doi:10.1016/j.jamda.2016.08.014
53. Mukherjee A, Biswas A, Roy A, *et al.* Behavioural and psychological symptoms of dementia: correlates and impact on caregiver distress. *Dement Geriatric Cogn Dis Extra* 2017;7:354-365. doi:10.1159/000481568
54. Bell CM, Araki SS, Neumann PJ. The association between caregiver burden and caregiver health-related quality of life in Alzheimer disease. *Alzheimer Dis Assoc Disord* 2001;15:129-36. doi: 10.1097/00002093-200107000-00004
55. Pinquart M, Sorensen S. Differences between caregivers and noncaregivers in psychological health and physical health: A meta-analysis. *Psychol Aging* 2003;18:250-67. doi: 10.1037/0882-7974.18.2.250
56. Vitaliano PP, Zhang J, Scanlan JM. Is caregiving hazardous to one's physical health? A meta-analysis. *Psychol Bull* 2003;129:946-72. doi:10.1037/0033-2909.129.6.946
57. Laudenslager ML."Anatomy of an Illness": Control from a caregiver's perspective. *Brain, Behavior, & Immunity* 2014;36:1–8. doi:10.1016/j.bbi.2013.08.012
58. Koerin BB, Harrigan MP. P.S I love you: Long-distance caregiving. *J Gerontol Soc Work* 2003;40:63-81. doi:10.1300/J083v40n01_05
59. Christie J, Smith GR, Williamson GM, *et al*. Quality of informal care is multidimensional. *Rehabil Psychol* 2009;54:173-81. doi:10.1037/a0015705
60. Smith GR, Williamson GM, Miller LS, *et al*. Depression and quality of informal care: A longitudinal investigation of caregiving stressors. *Psychol Aging* 2011;26:584-91. doi:10.1037/a0022263
61. Hodges LJ, Humphris GM, Macfarlane G. A meta-analytic investigation of the relationship between the psychological distress of cancer patients and their carers. *Soc Sci Med* 2005;60:1-12. doi:10.1016/j.socscimed.2004.04.018
62. Stall NM, Kim SJ, Hardacre KA, *et al*. Association of informal caregiver distress with health outcomes of community-dwelling dementia care recipients: a systematic review. *J Am Geriatr Soc* 2019;67:609-17. doi:10.1111/jgs.15690
63. Kuzuya M, Enoki H, Hasegawa J, *et al*. Impact of caregiver burden on adverse health outcomes in community-dwelling dependent older care recipients. *Am J Geriatr Psychiatry* 2011;19:382-91. doi:10.1097/JGP.0b013e3181e9b98d
64. Doran CM, Kinchin I. A review of the economic impact of mental illness. *Aust Health Rev* 2019;43:43-8. doi:10.1071/AH16115
65. Cooper C, Balamurali TBS, Livingston G. A systematic review of the prevalence and covariates of anxiety in caregivers of people with dementia. *Int Psychogeriatr* 2007;19:175-95. doi:10.1017/S1041610206004297
66. Bledsoe LK, Moore SE, Collins WL. Long distance caregiving: An evaluative review of the literature. *Ageing Int* 2010;*35*:293-310. doi:10.1007/s12126-010-9062-3
67. Benefield LE, Beck C. Reducing the distance in distance-caregiving by technology innovation. *Clin Interv Aging* 2007;2:267-72.
68. Shamseer L, Moher D, Clarke M, *et al*. Preferred reporting items for systematic review and meta-analysis protocols (PRISMA-P) 2015: elaboration and explanation. *BMJ* 2015;349:g7647. doi:10.1136/bmj.g7647
69. Lizarondo L, Stern C, Carrier J, *et al.* Chapter 8: Mixed methods systematic reviews. In: Aromataris E, Munn Z, eds. *Joanna Briggs Institute Reviewer's Manual*. The Joanna Briggs Institute 2017. https://wiki.joannabriggs.org/display/MANUAL/Chapter+8%3A+Mixed+methods+systematic+reviews (accessed 11 Jul 2020)

For peer review only - http://bmjopen.bmj.com/site/about/guidelines.xhtml

70. Shifren, KE. How caregiving affects development: Psychological implications for child, adolescent, and adult caregivers. Washington, DC: American Psychological Association 2009:1-223. doi:10.1037/11849-000
71. Rubira EA, Marcon SR, Belasco AGS, *et al*. Burden and quality of life of caregivers of children and adolescents with chemotherapy treatment for cancer. *Acta Paul Enferm* 2012;25:567-73. doi:10.1590/S0103-21002012005000020
72. Lyonette C, Yardley L. The influence on carer wellbeing of motivations to care for older people and the relationship with the care recipient. *Ageing Soc* 2003;23:487-506. doi:10.1017/S0144686X03001284
73. Abell, N. Assessing willingness to care for persons with AIDS: Validation of a new measure. *Res Soc Work Pract* 2001;11:118-30. doi:10.1177/104973150101100108
74. Radloff LS. The CES-D Scale: A Self-Report Depression Scale for Research in the General Population. *Appl Psychol Meas* 1977;1:385–401. doi:10.1177/014662167700100306
75. Zigmond AS, Snaith RP. The Hospital Anxiety and Depression Scale. *Acta Psychiatr Scand* 1983;67:361–70. doi:10.1111/j.1600-0447.1983.tb09716.x
76. McNair DM, Lorr M, Droppleman LF. Manual for the Profile of Mood States. San Diego, CA: Educational and Industrial Testing Services 1971.
77. Cohen S, Kamarck T, Mermelstein R. A Global Measure of Perceived Stress. *J Health Soc Behav* 1983;24:385–96. doi:10.2307/2136404
78. Zarit SH, Reever KE, Bach-Peterson J. Relatives of the impaired elderly: correlates of feelings of burden. *Gerontologist* 1980;20:649-55. doi:10.1093/geront/20.6.649
79. Cohen S, Hoberman HM. Positive events and social supports as buffers of life change stress. *Journal Appl Soc Psychol* 1983);13:99-125. doi:10.1111/j.1559-1816.1983.tb02325.x
80. Hong QN, Pluye P, Fàbregues S, *et al*. Mixed Methods Appraisal Tool (MMAT), version 2018. Canadian Intellectual Property Office, Industry Canada 2018:1-12.

For peer review only - http://bmjopen.bmj.com/site/about/guidelines.xhtml

Figure Legends

Figure 1

Flow diagram of the planned study selection process adapted from the Preferred Reporting Items for Systematic Reviews and Meta-Analyses (PRISMA) statement [68].

Figure 2

Qualitative synthesis of integrated "qualitised" quantitative data and qualitative data, following the convergent integrated approach as proposed by JBI methodology for mixed-method reviews [69].

For peer review only

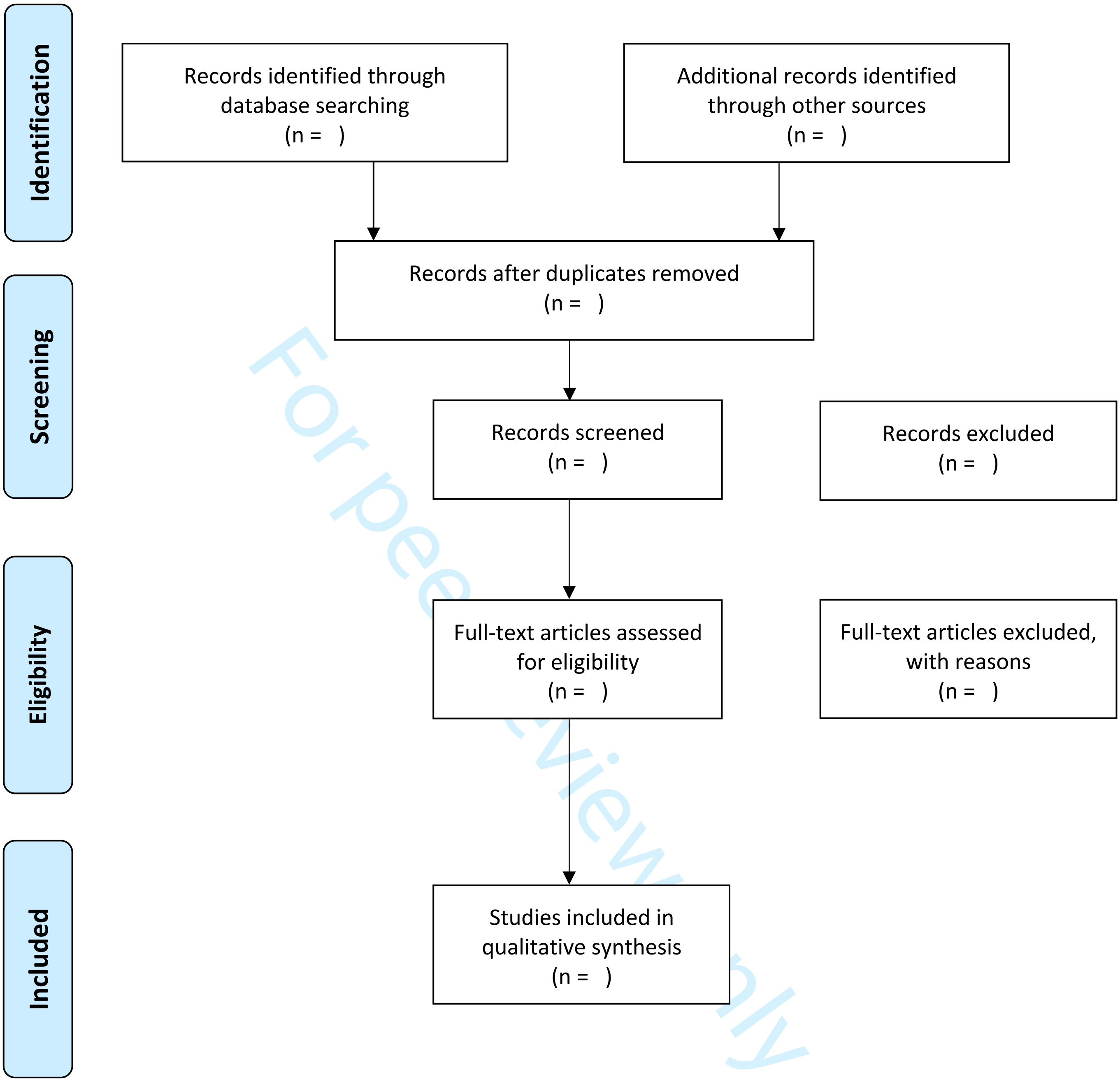
Identification
Records identified through database searching (n =)
Additional records identified through other sources (n =)
Records after duplicates removed (n =)
Screening
Records screened (n =)
Records excluded (n =)
Eligibility
Full-text articles assessed for eligibility (n =)
Full-text articles excluded, with reasons (n =)
Included
Studies included in qualitative synthesis (n =)

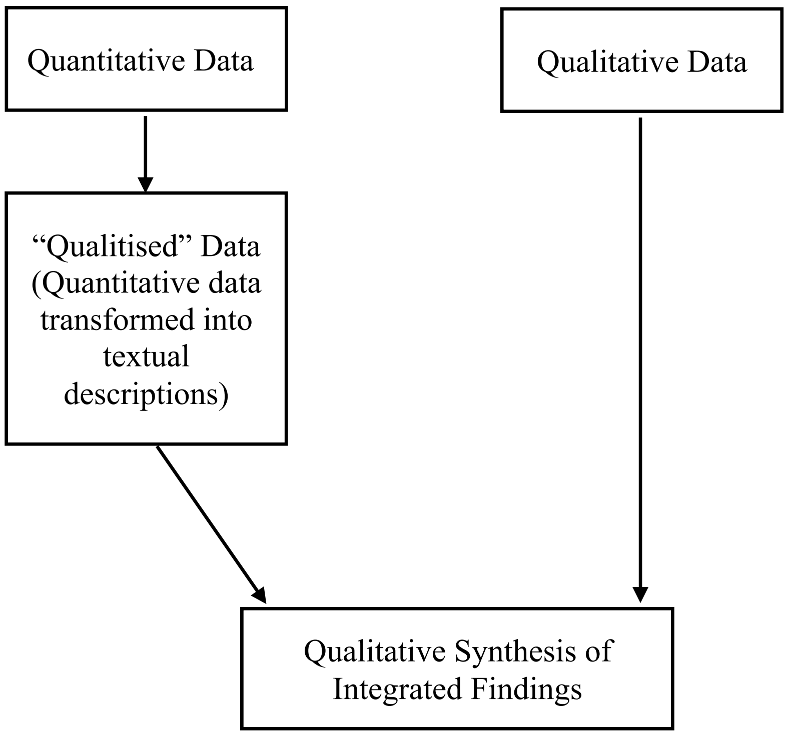
Quantitative Data
Qualitative Data
“Qualitised” Data (Quantitative data transformed into textual descriptions)
Qualitative Synthesis of Integrated Findings

view only

Motivations and willingness to provide care from a geographic distance, and the impact of distance care on caregivers' mental and physical health: A mixed-method systematic review protocol

PRISMA-P (Preferred Reporting Items for Systematic review and Meta-Analysis Protocols) 2015 checklist: recommended items to address in a systematic review protocol*

Section and topic	Item No	Checklist item	Location in text (page number)
**ADMINISTRATIVE INFORMATION**			
Title:			
Identification	1a	Identify the report as a protocol of a systematic review	1
Update	1b	If the protocol is for an update of a previous systematic review, identify as such	N/A
Registration	2	If registered, provide the name of the registry (such as PROSPERO) and registration number	8
Authors:			
Contact	3a	Provide name, institutional affiliation, e-mail address of all protocol authors; provide physical mailing address of corresponding author	1
Contributions	3b	Describe contributions of protocol authors and identify the guarantor of the review	13
Amendments	4	If the protocol represents an amendment of a previously completed or published protocol, identify as such and list changes; otherwise, state plan for documenting important protocol amendments	8
Support:			
Sources	5a	Indicate sources of financial or other support for the review	13
Sponsor	5b	Provide name for the review funder and/or sponsor	13
Role of sponsor or funder	5c	Describe roles of funder(s), sponsor(s), and/or institution(s), if any, in developing the protocol	13
**INTRODUCTION**			
Rationale	6	Describe the rationale for the review in the context of what is already known	3-7
Objectives	7	Provide an explicit statement of the question(s) the review will address with reference to participants, interventions, comparators, and outcomes (PICO)	7
**METHODS**			
Eligibility criteria	8	Specify the study characteristics (such as PICO, study design, setting, time frame) and report characteristics (such	8-10

For peer review only

		as years considered, language, publication status) to be used as criteria for eligibility for the review	
Information sources	9	Describe all intended information sources (such as electronic databases, contact with study authors, trial registers or other grey literature sources) with planned dates of coverage	10
Search strategy	10	Present draft of search strategy to be used for at least one electronic database, including planned limits, such that it could be repeated	10 & Supplementary file 2
Study records:			
Data management	11a	Describe the mechanism(s) that will be used to manage records and data throughout the review	10-11
Selection process	11b	State the process that will be used for selecting studies (such as two independent reviewers) through each phase of the review (that is, screening, eligibility and inclusion in meta-analysis)	10-11
Data collection process	11c	Describe planned method of extracting data from reports (such as piloting forms, done independently, in duplicate), any processes for obtaining and confirming data from investigators	11
Data items	12	List and define all variables for which data will be sought (such as PICO items, funding sources), any pre-planned data assumptions and simplifications	11 & Supplementary file 3
Outcomes and prioritization	13	List and define all outcomes for which data will be sought, including prioritization of main and additional outcomes, with rationale	9-10
Risk of bias in individual studies	14	Describe anticipated methods for assessing risk of bias of individual studies, including whether this will be done at the outcome or study level, or both; state how this information will be used in data synthesis	11
Data synthesis	15a	Describe criteria under which study data will be quantitatively synthesised	N/A
	15b	If data are appropriate for quantitative synthesis, describe planned summary measures, methods of handling data and methods of combining data from studies, including any planned exploration of consistency (such as I^2, Kendall's τ)	N/A
	15c	Describe any proposed additional analyses (such as sensitivity or subgroup analyses, meta-regression)	N/A
	15d	If quantitative synthesis is not appropriate, describe the type of summary planned	11-12 & Figure 2
Meta-bias(es)	16	Specify any planned assessment of meta-bias(es) (such as publication bias across studies, selective reporting within studies)	10-11
Confidence in cumulative evidence	17	Describe how the strength of the body of evidence will be assessed (such as GRADE)	N/A

N/A= Not Applicable

*** It is strongly recommended that this checklist be read in conjunction with the PRISMA-P Explanation and Elaboration (cite when available) for important clarification on the items. Amendments to a review protocol should be tracked and dated. The copyright for PRISMA-P (including checklist) is held by the PRISMA-P Group and is distributed under a Creative Commons Attribution Licence 4.0.**

From: Shamseer L, Moher D, Clarke M, Ghersi D, Liberati A, Petticrew M, Shekelle P, Stewart L, PRISMA-P Group. Preferred reporting items for systematic review and meta-analysis protocols (PRISMA-P) 2015: elaboration and explanation. BMJ. 2015 Jan 2;349(jan02 1):g7647.

For peer review only

Motivations and willingness to provide care from a geographic distance, and the impact of distance care on caregivers' mental and physical health: A mixed-method systematic review protocol

Supplementary File 2: Search Strategy – PubMed

#	Search Terms
#1	Distance caregiving [Title/Abstract] OR<br>Distance caregiver* [Title/Abstract] OR<br>Distant caregiving [Title/Abstract] OR<br>Distant caregiver* [Title/Abstract] OR<br>Distance carer* [Title/Abstract] OR<br>Distant carer* [Title/Abstract] OR<br>Spatial proximity + care [Title/Abstract] OR<br>Care from afar [Title/Abstract]] OR<br>Caregiving from afar [Title/Abstract] OR<br>Caregiver* from afar [Title/Abstract] OR<br>Proximate caregiver* [Title/Abstract] OR<br>Proximate caregiving [Title/Abstract] OR<br>Proximate carer* [Title/Abstract] OR<br>Out of town caregiver* [Title/Abstract] OR<br>Immigrant caregiver* [Title/Abstract] OR<br>Transnational caregiver* [Title/Abstract] OR<br>Transnational caregiving [Title/Abstract]

For peer review only

**#2**	motiv* [Title/Abstract] OR<br><br>motivation* to care [Title/Abstract] OR<br><br>motivation* to provide care [Title/Abstract] OR<br><br>oblig* [Title/Abstract] OR<br><br>filial [Title/Abstract] OR<br><br>duty [Title/Abstract] OR<br><br>guilt* [Title/Abstract] OR<br><br>reciproc* [Title/Abstract] OR<br><br>love [Title/Abstract] OR<br><br>willing* [Title/Abstract] OR<br><br>willingness to care [Title/Abstract] OR<br><br>willingness to provide care [Title/Abstract] OR<br><br>love [MeSH Terms]
**#3**	depress* [Title/Abstract] OR<br><br>distress [Title/Abstract] OR<br><br>stress [Title/Abstract] OR<br><br>mood [Title/Abstract] OR<br><br>anxiety [Title/Abstract] OR<br><br>well being [[Title/Abstract] OR<br><br>mental health [Title/Abstract] OR<br><br>caregiver burden [Title/Abstract] OR<br><br>burden [Title/Abstract] OR<br><br>physical symptoms [[Title/Abstract] OR<br><br>depression [MeSH terms] OR<br><br>anxiety [MeSH terms] OR<br><br>anxiety disorders [MeSH] OR<br><br>mood disorders [MeSH terms] OR

For peer review only

	mental health [MeSH terms] OR<br><br>health [MeSH terms]
**#4**	OR #2 / #3
**#5**	#1 AND #4

****Note.*** * = truncation symbol to identify variations and plurals of words

For peer review only

Motivations and willingness to provide care from a geographic distance, and the impact of distance care on caregivers' mental and physical health: A mixed-method systematic review protocol

Supplementary File 3: Data extraction form (Adapted from Lizarondo *et al.*) [69]

**Study Identification Features**	
**Reviewer**	
**Article Author(s)**	
**Study Title**	
**Date of Publication**	
**Publication Type**	• Research Article<br>- Journal Name:<br>- Record Number:<br>• PhD Thesis/Dissertation<br>• Conference Paper<br>• Other Please Specify: ___________
**Country of Study**	
**Research Aims and Objectives**	
**Methodology**	
**Type of Study (Quantitative, Qualitative or Mixed-method study)**	
**Methodology/Study Design (e.g. Cross-sectional study, Phenomenology)**	
**Method of Analysis (e.g. Thematic analysis, Hierarchical regression analyses)**	
**Method(s) of Data Collection (e.g. Online survey, Interviews, Focus groups)**	
**Inclusion/Exclusion Criteria**	
**Final Sample Size**	
**Recruitment Rate**	
**Drop-out Rate**	
**Time Points (If applicable)**	

For peer review only

**Drop-out Rate for Follow-up Points (If applicable)**		
**Participants' Characteristics**		
**Age**		*x̄ (SD)*
**Gender**		*%N*
**Ethnicity**		*%N*
**Care Recipient's Diagnosis**		
**Relationship to Care Recipient**		
**Time Caring**		
**Other Care Experience**		
**Employment Status**		
**Phenomena of Interest**		
**Concepts (C1) - Motivations to Care and/or Willingness to Care**	• Motivations to Care from a Geographic Distance<br>• Willingness to Care from a Geographic Distance<br>• Willingness to Perform Specific Care Tasks (e.g. emotional, nursing or instrumental care tasks)	
**Concepts (C2) – Mental and Physical Health Outcomes**	• Depression<br>• Anxiety<br>• Emotional Distress<br>• Caregiver Burden<br>• Perceived Physical Health<br>• Physical Symptoms	
**Context-related Information**		
**Geographic Distance from the Care Recipient (e.g. assessed using miles or travel time)**		
**Quantitative Findings**		
**Measures**		
**Key Findings**		
**Qualitative Findings**		
**Identified Themes or Subthemes**		
**Author's Conclusions**		
**Reviewer's Comments**		

BMJ Open

Motivations and willingness to provide care from a geographic distance, and the impact of distance care on caregivers' mental and physical health: A mixed-method systematic review protocol

Journal:	*BMJ Open*
Manuscript ID	bmjopen-2020-045660.R1
Article Type:	Protocol
Date Submitted by the Author:	07-Mar-2021
Complete List of Authors:	Bei, Eva; Bar-Ilan University, Department of Psychology<br>Zarzycki, Mikołaj; Bangor University, School of Psychology<br>Morrison, Valerie; Bangor University, School of Psychology<br>Vilchinsky, Noa; Bar-Ilan University, Department of Psychology
**Primary Subject Heading**:	Public health
Secondary Subject Heading:	Public health, Mental health
Keywords:	PRIMARY CARE, MENTAL HEALTH, Depression & mood disorders < PSYCHIATRY, Anxiety disorders < PSYCHIATRY

SCHOLARONE™
Manuscripts

I, the Submitting Author has the right to grant and does grant on behalf of all authors of the Work (as defined in the below author licence), an exclusive licence and/or a non-exclusive licence for contributions from authors who are: i) UK Crown employees; ii) where BMJ has agreed a CC-BY licence shall apply, and/or iii) in accordance with the terms applicable for US Federal Government officers or employees acting as part of their official duties; on a worldwide, perpetual, irrevocable, royalty-free basis to BMJ Publishing Group Ltd ("BMJ") its licensees and where the relevant Journal is co-owned by BMJ to the co-owners of the Journal, to publish the Work in this journal and any other BMJ products and to exploit all rights, as set out in our licence.

The Submitting Author accepts and understands that any supply made under these terms is made by BMJ to the Submitting Author unless you are acting as an employee on behalf of your employer or a postgraduate student of an affiliated institution which is paying any applicable article publishing charge ("APC") for Open Access articles. Where the Submitting Author wishes to make the Work available on an Open Access basis (and intends to pay the relevant APC), the terms of reuse of such Open Access shall be governed by a Creative Commons licence – details of these licences and which Creative Commons licence will apply to this Work are set out in our licence referred to above.

Other than as permitted in any relevant BMJ Author's Self Archiving Policies, I confirm this Work has not been accepted for publication elsewhere, is not being considered for publication elsewhere and does not duplicate material already published. I confirm all authors consent to publication of this Work and authorise the granting of this licence.

For peer review only

TITLE

Motivations and willingness to provide care from a geographic distance, and the impact of distance care on caregivers' mental and physical health: A mixed-method systematic review protocol

AUTHORS

Eva Bei (corresponding author), Department of Psychology, Faculty of Social Sciences, Bar-Ilan University, Ramat Gan, 5290002, Israel; ORCID ID: 0000-0002-3093-0829; Email: eva.bei@biu.ac.il; Telephone: +972 0559455802

Mikołaj Zarzycki, School of Psychology, Bangor University, Bangor, Gwynedd, UK; ORCID ID: 0000-0002-8357-563X; Email: m.zarzycki@bangor.ac.uk

Val Morrison, School of Psychology, Bangor University, Bangor, Gwynedd, UK; ORCID ID: 0000-0002-4308-8976; Email: v.morrison@bangor.ac.uk

Noa Vilchinsky, Department of Psychology, Faculty of Social Sciences, Bar-Ilan University, Ramat Gan, 5290002, Israel; ORCID ID: 0000-0003-4965-4745; Email: noa.vilchinsky@biu.ac.il

WORD COUNT: 3649

For peer review only

ABSTRACT

Introduction

Distance Caregivers (DCGs) are a growing population with substantial contribution to informal care. While a reasonable amount is known on the determinants of motives and willingness to provide local informal care, and the local caregiver outcomes, reports for the distance caregiving population are lacking. An evidence synthesis of what motivates and makes DCGs willing to care from a distance and the impact of that care on their mental and physical health, would highlight any gaps or consensus in knowledge. This would guide the research needed towards the development of tailored interventions, in order to support DCGs and promote the sustainability of distance care.

Methods and analysis

This protocol adheres to Preferred Items for Reporting of Systematic Reviews and Meta-Analyses Protocols (PRISMA) guidelines and the Joanna Briggs Institute (JBI) Methodology for mixed-method reviews. A comprehensive search strategy will be conducted in four electronic databases (CINAHL, MEDLINE, PubMed and PsycINFO). Grey literature will be also assessed to minimise publication bias. Two independent reviewers will assess each study for inclusion and any discrepancies will be resolved with the consultation of a third reviewer. Eligible studies for inclusion will be English language studies exploring the motives and willingness to care for a care recipient with a chronic disease, disability or frailty from a geographic distance; or studies focusing on the mental and physical health outcomes of DCGs. Qualitative and quantitative data will be integrated in a single qualitative synthesis following the JBI convergent integrated approach. Study quality will be assessed using the Mixed Methods Appraisal Tool (MMAT) version 2018.

Ethics and dissemination

Ethical approval is not required for this study as no primary data will be collected. Findings will be disseminated through peer-reviewed publication and presentations at academic conferences and lay summaries for various stakeholders.

PROSPERO registration number CRD42020156350.

STRENGTHS AND LIMITATIONS OF THIS STUDY

- The review will set the groundwork and inform research needed to guide the development of tailored interventions to support DCGs and promote the sustainability of distance care.
- The mixed-method design of the study will ensure a wide variety of data on distance care is captured from both qualitative and quantitative research findings.
- A rigorous, systematic approach will be applied to searching, screening, extracting and analysing evidence in four different academic databases and in grey literature.
- Anticipated potential limitations include high degree of heterogeneity across studies as there is no consensus on the definition of distance caregiving which makes it difficult to compare studies.
- The review will be restricted to studies published only in English, which may cause language bias.

KEY WORDS

Distance caregiving, geographic distance, motivations to care, willingness to care, mental health, physical health

INTRODUCTION

With the ageing of the population worldwide, numbers of older and care-dependent adults are rising [1-5]. Informal caregivers who provide unpaid care to a family member or friend with long-term care needs, are an essential pillar for sustaining healthcare systems worldwide and maintaining outpatient care [6-8]. Traditional patterns of informal care usually involved family or friends co-residing or living close to the care recipient [9]. However, as the demand for caregivers continues to grow, social changes such as urbanisation, gendered roles within society, increased labor market mobility and globalisation have affected the traditional ways of providing care [9-11]. Distance care has emerged in those cases where adult caregivers are not staying with their care recipient but making efforts for providing informal care from a geographic distance [11-13].

It is estimated that 15–20% of all informal care is provided by Distance Caregivers (DCGs) [12]. One of the problems in this field of research is that there is no consensus on how to best define distance caregiving. Most research to date has used mileage [12] or travel time categories [14,15] to measure the geographic distance from the care recipient and define

For peer review only - http://bmjopen.bmj.com/site/about/guidelines.xhtml

DCGs. Overall, travel time and space along with a number of socioeconomic factors which are confounded with distance, such as travel costs and access to transportation, will influence distance caregiving - in its existence as well as in its extent [16].

In their previous review, Cagle and Munn [17] suggested that the definition of distance care as articulated by Parker *et al.* [18], is the most comprehensive and compelling. Indeed, the definition appears to be appropriate as it operationalises geographic distance without focusing exclusively on distance and travel time but also on a number of other socioeconomic factors that determine geographic distance and affect distance care provision. According to Parker *et al.*, a DCG could be defined as: "Anyone (1) who provides informal, unpaid care to a person experiencing some degree of physical, mental, emotional, or economic impairment that limits independence and necessitates assistance; and (2) who experiences caregiving complications because of geographic distances from the recipient, as determined by distance, travel time, travel cost, personal mobility problems, limited transportation, and other related factors that affect the caregiver's access to the care recipient" [18].

Demographic analyses show that most DCGs are adult children providing assistance to a family member, usually an aged parent, parent in-law or step-parent with a chronic health condition, disability or other long-lasting healthcare needs [17,19]. DCGs engage in many activities to ensure that the needs of their care recipient are met, such as providing social and emotional support, managing financial affairs, monitoring and coordinating care and healthcare services [12, 20-22]. Although most DCGs are unable to provide hands-on care on a daily basis as local caregivers, they are significantly involved in performing practical and nursing tasks when visiting their loved one. In fact, approximately 75% provide assistance with Instrumental Activities of Daily Living (IADLs) such as household tasks, cooking, grocery shopping, medical care and transportation [12]. Also, 40% report assisting with Activities of Daily Living (ADLs) such as bathing, dressing, feeding and toileting [12].

Motivations and willingness to provide informal care

In recent years, a growing body of literature has explored caregivers' motivations and willingness to carry out a caregiving role [23-25]. Research on local caregiving has identified multiple and often inter-related determinants of caregivers' motives and willingness to care for a loved one [23]. Informal care is likely to be provided out of love and affection, reflecting a long-lasting family or friend relationship between the caregiver and the care recipient [26,27]. Reciprocity is also a frequently cited motive for informal care provision.

For peer review only - http://bmjopen.bmj.com/site/about/guidelines.xhtml

Adult children or children in-law often reciprocate the care and love received in the past from the care recipient, by undertaking the caregiving role [28-32]. In addition, caregiving is frequently described as a family duty, filial obligation or responsibility. Adult children are often motivated to care as a means of fulfilling their care responsibilities, in socio-cultural contexts where caregiving is viewed as an unspoken family value, a moral obligation [28, 33-37]. Beside these factors, availability of other caregivers [30,34] and resources for formal care [38] may also have an effect on the provision of informal care.

Baldassar and colleagues [21] demonstrated that DCGs may describe some similar motives to care to those of local caregivers. Despite the unique challenges and complications caused by physical separation, DCGs felt that they were greatly indebted to their parents for raising them and it was now their turn to reciprocate the care received and pay them back even from a distance [21]. They also reported a strong sense of obligation to care based on moral and societal expectations regarding caretaking duties [21]. However, DCGs may also report motives to care unique to the distance caregiving situation, for example, in a recent study [39], DCGs expressed feelings of guilt about not being physically present to fulfil their duty to care. Although physical distance limited DCGs from providing care on a regular basis, it also worked as a strong motivator, increasing their willingness and efforts for providing adequate care from afar [39].

Previous findings have highlighted the significance of motivations and willingness to take on the caregiving role. Camden and colleagues [40] found that those who were unwilling to provide care, reported higher abusive behaviors towards the care recipient while their loved one was more likely to be admitted to a care home the following year. Another qualitative study showed that caregivers who provided care out of societal expectations had less control over the caregiving challenges [41]. Additionally, Romero-Moreno *et al.* [24] reported that extrinsic motives for caring (i.e. filial obligation) are associated with poorer caregivers' mental health. Similarly, in their systematic review, Quinn *et al*. [25] found that caregivers motives to provide informal care can have implications on their psychological well-being.

Mental and physical health outcomes of informal caregiving

Studies of broader caregiving samples suggest that caregivers can experience both positive and negative outcomes as a result of their caretaking responsibilities [42-44]. The positive aspects of caregiving may include an enhanced relationship with the care recipient, feelings of personal growth, gains and satisfaction, and an overall rewarding and meaningful

For peer review only - http://bmjopen.bmj.com/site/about/guidelines.xhtml

experience [8,42,45-47]. However, caregivers may also experience negative outcomes related to the caregiving role, such as poor mental and physical health [47-48]. Numerous studies have identified high levels of caregiver burden across a wide range of chronic diseases, as a multidimensional response to the various stressors associated with caring [48-50]. In terms of psychological morbidity, high levels of emotional distress and increased symptoms of anxiety and depression have been reported [51-53] with, for example, a meta-analytical review reporting a prevalence of anxiety and depressive symptomatology among caregivers of stroke survivors as above 20% and 40%, respectively [52]. In addition, many caregivers experience poor physical health, related to their role [54-56]. A meta-analysis of 84 studies conducted across a range of health conditions revealed that caregivers reported statistically significant lower levels of well-being and physical health than non-caregivers [55], with some specific findings pointing to caregivers showing poorer immune response [57] and exhibiting greater cardiovascular reactivity [56] than non-caregivers.

Geographic distance creates additional burdens for what is already often a stressful care work [17]. Koerin and colleagues [58] found that almost 80% of DCGs reported emotional distress related to feelings of inadequacy on how to assess the needs of their loved one from afar and uncertainty regarding the progression of their illness. Additionally, in their recent study, Li *et al.* [14] found that adult child primary caregivers who live more than 30 minutes away from their frail older parents had higher levels of depression than co-residing caregivers. Findings also showed that although co-residing caregivers were more distressed by objective caregiver burden, DCGs were more distressed by subjective caregiver burden which includes perceptions and attitudes related to caregiving [14].

The negative mental and physical health outcomes of caregiving can threaten caregivers' commitment to the welfare of the care recipient and reduce the quality of care provision [59,60]. Additionally, poor caregiver outcomes are associated with adverse health outcomes for care recipients, including higher levels of emotional distress, elder abuse, mortality and hospitalization [61-63]. Increased morbidity of caregivers can also lead to high individual and societal burdens and affect the sustainability of informal care [63,64].

For peer review only

Aim of the review

DCGs are a growing population who make a substantial contribution to informal care. To-date, to the best of our knowledge, no review has systematically evaluated, and quality appraised the current evidence on motivations and willingness to care from a distance, and the impact of distance care on caregivers' mental and physical health outcomes. Most systematic reviews have currently focused on local caregivers [23,24,65], excluding the distance caregiving population. In addition, past reviews of distance caregiving have been limited to evaluating evidence on the phenomenon of distance care in general, focusing on the definition and sociodemographic characteristics of DCGs and describing only briefly the benefits and costs of such a role [17,66]. Other reviews have exclusively focused on distance caregiving for patients with advanced cancer without exploring distance care on other health conditions with different care needs or demands [13]; or on the availability of technology-based and eHealth interventions to support DCGs [67].

However, as previously stated, DCGs may report unique experiences and motives to care, associated with the geographic and spatial distance from the care recipient [21,39]. Experiencing the added challenges and complications of caring from afar, DCGs may also be at higher risk of poor mental and physical health outcomes [14,17,58]. A systematic review would synthesise evidence from studies on what motivates DCGs and makes them willing to provide care from a distance, and what is the impact of that care on their mental and physical health. Further understanding of these issues, would provide evidence for the development of geographically sensible and tailored interventions in order to promote the sustainability of distance care and support those caring from afar. Therefore, this review aims to:

1) Synthesise and critique the evidence on the determinants of motivations and willingness to care from a geographic distance.
2) Synthesise and critique the evidence on the impact of distance caregiving on caregivers' mental and physical health.

For peer review only - http://bmjopen.bmj.com/site/about/guidelines.xhtml

METHODS AND ANALYSIS

This protocol is guided by the Preferred Items for Reporting of Systematic Reviews and Meta-Analyses Protocols (PRISMA) checklist [68] (Online supplementary appendix 1) and the Joanna Briggs Institute Methodology for Mixed-Methods Systematic Reviews [69].

Study registration

Based on the PRISMA guidelines [68], the protocol for this systematic review was registered on the international database of prospectively registered systematic reviews in health and social care, PROSPERO (registration number: CRD42020156350). Any important protocol amendments will be recorded in PROSPERO and published with the results of the review.

Eligibility criteria

Types of studies

Eligible studies for inclusion will be English language peer-reviewed studies or unpublished studies such as doctoral theses in order to reduce publication bias. These will include quantitative studies (analytical observational and descriptive observational studies), studies that focus on qualitative data and mixed-method study designs. The inclusion of different types of research will provide more informative findings for each of the two review objectives and increase the ability of those findings to inform future research, policy and practice.

Participants

The population of interest are adult DCGs (aged 18 and above) of adult family members or friends with healthcare needs. The comprehensive definition of distance care by Parker *et al.* [18] was used and adapted for the purposes of this review, and in order to maximize our access to empirical studies that conceptualised geographic distance from the care recipient in different ways. Thus, as a DCG could be defined:

(1) Anyone who provides informal, unpaid care to a relative or friend with a chronic illness, disability or frailty that limits independence and necessitates assistance;
AND

(2) who experiences caregiving complications because of geographic distances from the care recipient, as determined by distance, travel time, travel cost, personal mobility problems, limited transportation, or other related factors that affect the caregiver's access to the care recipient.

For peer review only - http://bmjopen.bmj.com/site/about/guidelines.xhtml

Care recipient's health conditions eligible for this review will include any chronic illness and disability (e.g. cancer, dementia, cardiovascular disease, stroke etc.) or simply frailty. Studies exclusively focusing on young DCGs (under 18 years old) or DCGs of children and adolescents will be excluded, as young caregiving [70] and care receiving [71] experiences are associated with additional needs and burdens.

Outcomes

Primary outcomes

The primary outcomes of interest are caregivers' motives and willingness to provide care from a geographic distance. These may include qualitative and/or quantitative data. Qualitative findings describing various determinants of motives and willingness to care from a distance such as for example family values, love and affection, reciprocity, guilty feelings, filial obligation and specific illness characteristics will be reported. In addition, qualitative studies exploring willingness to perform a variety of caregiving tasks from afar or when visiting the care recipient, such as emotional, nursing and instrumental care tasks, will be considered. For quantitative findings, studies must report on motives and willingness to care from a distance, assessed using a validated self-report instrument (e.g. Motivations in Elder Care Scale [72], Willingness to Care Scale [73]) or a self-report instrument developed for the purposes of the included study. Given that many studies explored the determinants of motives and willingness to provide local care using qualitative methodology, it is envisaged that our outcomes will be also primarily explored through qualitative data.

Secondary outcomes

Secondary outcomes of interest are those studies assessing the impact of distance care provision on caregivers' mental and physical health outcomes, specifically depression, anxiety, emotional distress, caregiver burden, perceived physical health or physical symptoms. Eligible studies for inclusion will be quantitative studies that assessed distance caregiver outcomes using a validated self-report outcome instrument or an instrument developed for the purposes of the included study. Examples of validated psychrometric instruments for the secondary outcomes of interest include the Centre for Epidemiologic Studies-Depression Scale (CES-D) [74], the Hospital Anxiety and Depression Scale (HADS) [75], the Profile of Mood States (POMS) [76], the Perceived Stress Scale [77], the Zarit Burden Interview [78] or the Cohen-Hoberman Inventory of Physical Symptoms (CHIPS)

For peer review only

[79]. Qualitative findings exploring manifestations and perceptions of emotional distress, anxiety, depression, burden and physical health will be also considered.

Search strategy

A detailed search strategy without limits on publication date, will be conducted on four relevant electronic databases (CINAHL, MEDLINE, PubMed and PsycINFO), to comprehensively search for studies on distance caregiving. Grey literature, including the OpenGrey (http://www.opengrey.eu/) and Electronic Theses Online Service (ETHOS; https://ethos.bl.uk/) databases, will be also searched to maximize our access to potentially relevant studies and reduce publication bias. In addition, the reference lists of all studies included will be screened to identify additional citations of interest.

The search strategy was developed using key terms related to the distance caregiving population and the primary and secondary outcomes of interest. The search was also informed by past reviews on distance care [13,17]. A variation of controlled vocabulary, relevant Medical Subject Headings (MeSH) when possible and free-text terms contained in the title/abstracts of publications will be applied. An example of the search strategy string terms used for PubMed database is presented (Online supplementary appendix 2). Search strategy string will be then adapted to the remaining three electronic databases. Per database these terms will be mapped to Subject Headings. Included studies will be restricted to those written in English for ease of interpretation.

Study Selection

Following the search, all identified citations will be imported into Zotero and duplicates removed. In the first stage of primary screening, one reviewer (EB) will screen the titles of each study based on the inclusion criteria and when these are deemed relevant, she will go through abstracts. In addition, a second author (NV) will be consulted on the primary screening process using the eligibility criteria, and any doubts or conflicts for the titles or abstracts that are deemed relevant will be resolved through discussion. Once narrowed down by abstracts, a full-text review process will be completed in duplicate by two reviewers (EB and MZ) for studies that met the eligibility criteria at screening and for studies with unclear relevance. A third independent author (NV) will be consulted to resolve any discrepancies that arose between the two reviewers during the full-text process. Additional records identified from searches of grey literature and reference lists will be also assessed in detail against the eligibility criteria by the principal researcher (EB). Original authors of studies

For peer review only - http://bmjopen.bmj.com/site/about/guidelines.xhtml

identified will be contacted if the full-text paper was not available or the relevance of a paper was unclear. The PRISMA flow diagram will outline the process of study selection (Figure 1) [68]. Reasons for exclusion of full-text studies that do not meet the eligibility criteria will be also recorded in the flow diagram.

Figure 1

Assessment of methodological quality

To assess the methodological quality of the included studies, the Mixed Methods Appraisal Tool (MMAT) version 2018 will be used, a quality appraisal instrument for mixed-method reviews [80]. The MMAT has been content validated and piloted across all methodologies [80]. The instrument consists of two screening questions for all study designs and five sections of specific questions regarding study type. The quality of each study will be assessed as low (0-1/5 criteria met), medium (2-3/5 criteria met) or high (4-5/5 criteria met), using the total number of MMAT criteria that were met. Quality appraisal will be completed in duplicate by two independent reviewers (EB and MZ). Any discrepancies will be resolved with the consultation of a third reviewer (VM). The strength of the body of evidence will be discussed and narratively incorporated into the synthesis. No study will be excluded on the basis of their methodological quality, as they could still offer valuable insight. Yet, findings of the studies assessed as low will be interpreted with caution.

Data Extraction

Data extraction process will be carried out using the JBI data extraction tool [69] as adapted and modified for the purposes of this review (Online supplementary appendix 3). Data will be extracted for each study by one reviewer (EB) and independently extracted by the second reviewer (MZ) for accuracy, employing a double-coding on portion of 80% of the included studies. Any discrepancies will be resolved with the consultation of a third reviewer (NV). Data extracted from the included studies will comprise research aims and objectives, study methods, population characteristics, phenomena of interest and context-related information, main study findings and relevant outcomes. Extracted information will vary across different study designs.

For peer review only - http://bmjopen.bmj.com/site/about/guidelines.xhtml

Data synthesis

Data synthesis will involve a convergent integrated approach, as per JBI methodology for questions that can be addressed by both quantitative and qualitative research designs (Figure 2) [69]. Extracted data from quantitative findings will be first converted into "qualitised data". This will involve a narrative interpretation of the quantitative results and the transformation of numerical data into textual descriptions. According to the JBI guidelines, "qualitising" quantitative data is recommended, as codifying quantitative data is less error-prone than attributing numerical values to extracted data of qualitative studies. At the simplest level, "qualitised" data will comprise the description of sample based on descriptive statistics such as average or percentage scores. For quantitative data with a temporal or longitudinal component and those that explore associations using inferential statistics, "qualitising process" will involve the identification of the variables included in the data analysis using textual descriptions and numerical data transformation. The "qualitised" data will be then assembled and pooled with the results of qualitative studies. Similar to the meta-aggregative approach for JBI qualitative reviews, a detailed examination of the pooled data will be finally undertaken to identify categories based on similarity and produce a set of integrated findings for each of the two review objectives [69].

Figure 2

PATIENT AND PUBLIC INVOLVEMENT

Patients and members of the public were not involved in the design and development of this protocol.

ETHICS AND DISSEMINATION

Ethical approval and consent to participate are not required for the proposed systematic review as no primary data will be collected. Findings will be disseminated through publication in an international peer reviewed journal and presented at local and international scientific conferences on informal care and health psychology. A lay summary of the review will be written for healthcare organisations and non-scientific audiences such as caregivers and their care recipients and disseminated through mass emails, social media, blogs and appropriate webpages.

For peer review only - http://bmjopen.bmj.com/site/about/guidelines.xhtml

AUTHORS' CONTRIBUTIONS

EB contributed to the conception and design of the study and wrote the manuscript. NV contributed to the conception and design of the study and critically revised the manuscript draft. MZ and VM critically revised the study design and the manuscript draft. All authors approved the final manuscript.

FUNDING

This work is funded by the European Union's Horizon 2020 research and innovation programme under the Marie-Sklodowska Curie grant agreement no 814072.

DISCLAIMER

The funder had no role in this protocol's development.

COMPETING INTERESTS

None declared.

PATIENT CONSENT FOR PUBLICATION

Not required.

ABBREVIATIONS

CINAHL: Cumulative Index to Nursing and Allied Health Literature
PRISMA-P: Preferred Reporting Items for Systematic Reviews and Meta-Analysis Protocols
PROSPERO: International prospective register of systematic reviews

For peer review only - http://bmjopen.bmj.com/site/about/guidelines.xhtml

REFERENCES

1. Casal BR, Rivera BC, Currais LN. Alzheimer's disease and the quality of life of the informal caregiver. *Rev Esp Geriatr Gerontol* 2019;54:81-7. doi:10.1016/j.regg.2018.10.008
2. Li J, Han X, Zhang X, *et al*. Spatiotemporal evolution of global population ageing from 1960 to 2017. *BMC public health* 2019; 19:127. doi:10.1186/s12889-019-6465-2
3. Sanai SA, Saini V, Benedict RH, *et al*. Aging and multiple sclerosis. *Mult Scler J* 2016;22:717-25. doi:10.1177/1352458516634871
4. Spasova S, Baeten R, Coster S, *et al*. *Challenges in long-term care in Europe: A study of national policies*. Brussels: European Comission: European Social Policy Network (ESPN), 2018.
5. Verbakel E. How to understand informal caregiving patterns in Europe? The role of formal long-term care provisions and family care norms. *Scand J Public Healt 2018*;46:436-47.
6. Colombo F, Llena-Nozal A, Mercier J, *et al*. Help wanted? providing and paying for long-term care. OECD Publishing 2011. doi:10.1787/9789264097759-en
7. Revenson T, Griva K, Luszczynska, *et* al. Caregiving in the illness context. Hampshire: Springer 2016. doi:10.1057/9781137558985
8. Roth DL, Fredman L, Haley WE. Informal caregiving and its impact on health: A reappraisal from population-based studies. *Gerontologist* 2015;55:309-19. doi: 10.1093/geront/gnu177
9. Baldassar L, Baldock CV, Wilding R. Families caring across borders: Migration, ageing and transnational caregiving. Springer 2006.
10. Croucher K, Gilroy R, Bevan M, *et al*. The mobilities of care in later life: exploring the relationship between caring and mobility in the lives of older people. *Ageing Soc* 2020;1-22. doi:10.1017/S0144686X20000100
11. Kaushik A. Elder Care from a Distance: Emerging Trends and Challenges in the Contemporary India. In: Shankardass M. Ageing Issues and Responses in India. Springer, Singapore 2020:97-113. doi: 10.1007/978-981-15-5187-1_6
12. National Alliance for Caregiving. Miles away: The MetLife study of long-distance caregiving. New York, NY: MetLife Mature Market Institute 2004:1-13.
13. Douglas SL, Mazanec P, Lipson A, *et al*. Distance caregiving a family member with cancer: A review of the literature on distance caregiving and recommendations for future research. *World J Clin Oncol* 2016;7:214. doi:10.5306/wjco.v7.i2.214
14. Li M, Mao W, Chi I, *et al*. Geographical proximity and depressive symptoms among adult child caregivers: social support as a moderator. *Aging Ment Health* 2019;23:205-13. doi: 10.1080/13607863.2017.1399349
15. Roff LL, Martin SS, Jennings LK., *et al*. Long distance parental caregivers' experiences with siblings: A qualitative study. *Qual Soc Work* 2007;6:315–34. doi:10.1177/ 1473325007080404
16. Bei E, Rotem-Mindali O, Vilchinsky N. Providing Care From Afar: A Growing Yet Understudied Phenomenon in the Caregiving Field. *Front Psychol* 2020;11:681. doi: 10.3389/fpsyg.2020.00681
17. Cagle JG, Munn JC. Long-distance caregiving: a systematic review of the literature. *J Gerontol Soc Work* 2012;55:682-707. doi:10.1080/01634372.2012.703763

For peer review only - http://bmjopen.bmj.com/site/about/guidelines.xhtml

18. Parker MW, Church W, Toseland RW. Caregiving at a Distance. In: Berkman B, D'Ambruoso S, eds. Handbook of Social Work in Health and Aging. New York: Oxford University Press 2006: 391-406. doi:10.1093/acprof:oso/9780195173727.003.0033
19. Mazanec P. Distance caregiving a parent with cancer. *Semin Oncol Nurs* 2012;28:271-78. doi: 10.1016/ j.soncn.2012.09.010
20. Harrigan MP, Koerin BB. Long distance caregiving: Personal realities and practice implications. *Reflections (Long Beach)* 2007; 13:5–16.
21. Baldassar L. Transnational families and aged care: The mobility of care and the migrancy of ageing. *J Ethn Migr Stud* 2007;33:275-97. doi:10.1080/13691830601154252
22. Baldock CV. Migrants and their parents: Caregiving from a distance. *J Fam Issues* 2000;21:205-24. doi: 10.1177/019251300021002004
23. Greenwood N, Smith R. Motivations for being informal carers of people living with dementia: a systematic review of qualitative literature. *BMC Geriatr* 2019;19:169. doi:10.1186/s12877-019-1185-0
24. Romero-Moreno R, Márquez-González M, Losada A, *et al*. Motives for caring: relationship to stress and coping dimensions. *Int Psychogeriatr* 2011,23:573. doi:10.1017/S1041610210001821
25. Quinn C, Clare L, Woods RT. The impact of motivations and meanings on the well-being of caregivers of people with dementia: a systematic review. *Int Psychogeriatr* 2010; 22:43–55. doi:10.1017/S1041610209990810
26. Lin MC, Macmillan M, Brown N. A grounded theory longitudinal study of carers' experiences of caring for people with dementia. *Dementia* 2011;11:181–97. doi: 10.1177/1471301211421362
27. McDonnell E, Ryan AA. The experience of sons caring for a parent with dementia. *Dementia*. 2014;13:788–802. doi: 10.1177/1471301213485374
28. Donorfio LK, Kellett K. Filial responsibility and transitions involved: A qualitative exploration of caregiving daughters and frail mothers. *J Adult Dev* 2006;13:158-67. doi:10.1007/s10804-007-9025-4
29. Qadir F, Gulzar W, Haqqani S, *et al*. A pilot study examining the awareness, attitude, and burden of informal caregivers of patients with dementia. *Care Manag J* 2013;14:230–40. doi: 10.1891/1521-0987.14.4.230
30. Quinn C, Clare L, Woods RT. Balancing needs: The role of motivations, meanings and relationship dynamics in the experience of informal caregivers of people with dementia. *Dementia* 2015;14:220-37. doi:10.1177/1471301213495863
31. Santos RL, Sousa MF, Ganem AC, *et al*. Cultural aspects in dementia: differences in the awareness of Brazilian caregivers. *Trends Psychiatry Psychother* 2013; 35:191-7. doi: 10.1590/s2237-60892013000300006
32. van Wezel N, Francke AL, Kayan-Acun E, *et al*. Family care for immigrants with dementia: the perspectives of female family carers living in the Netherlands. *Dementia* 2016;15:69–84. doi: 10.1177/1471301213517703
33. Dunér A. Motives, experiences and strategies of next of kin helping older relatives in the Swedish welfare context: a qualitative study. *Int J Soc Welf* 2010;19:54-62. doi: 10.1111/j.1468-2397.2008.00631.x
34. Parveen S, Morrison V, Robinson CA. Ethnicity, familism and willingness to care: Important influences on caregiver mood? *Aging Ment Health* 2013;17:115-24. doi:10.1080/13607863.2012.717251
35. Parveen S, Morrison V, Robinson CA. Ethnic variations in the caregiver role: A qualitative study. *J Health Psychol* 2011;16:862-72. doi: 10.1177/1359105310392416

For peer review only - http://bmjopen.bmj.com/site/about/guidelines.xhtml

36. Kim Y. Korean American family post caregivers on dementia caregiving: a phenomenological inquiry. *J Gerontol Soc Work* 2009;52:600–17. doi: 10.1080/01634370903048352
37. Yoo GJ, Kim BW. Remembering sacrifices: Attitude and beliefs among second-generation Korean Americans regarding family support. *J Cross Cult Gerontol* 2010;25:165-81. doi:10.1007/s10823-010-9116-8
38. Chang YP, Schneider JK, Sessanna L. Decisional conflict among Chinese family caregivers regarding nursing home placement of older adults with dementia. *J Aging Stud* 2017;25:436–44. doi:10.1016/j.jaging.2011.05.001
39. Amin I, Ingman S. Eldercare in the transnational setting: Insights from Bangladeshi transnational families in the United States. *J Cross Cult Gerontol* 2014;29:315-28. doi:10.1007/s10823-014-9236-7
40. Camden A, Livingston G, Cooper C. Reasons why family members become carers and the outcome for the person with dementia: results from the CARD study. *Int Psychogeriatr* 2011;23:1442. doi:10.1017/S1041610211001189
41. Ng HY, Griva K, Lim HA, *et al*. The burden of filial piety: A qualitative study on caregiving motivations amongst family caregivers of patients with cancer in Singapore. *Psychol Health* 2016;31:1293-310. doi:10.1080/08870446.2016.1204450
42. Li Q, Loke AY. The positive aspects of caregiving for cancer patients: a critical review of the literature and directions for future research. *Psycho-Oncol* 2013;22:2399-407. doi:10.1002/pon.3311
43. Lambert SD, Levesque JV, Girgis A. The impact of cancer and chronic conditions on caregivers and family members. In: Koczwara. Cancer and chronic conditions: Addressing the Problem of Multimorbidity in Cancer Patients and Survivors Springer, Singapore 2016:159-202. doi:10.1007/978-981-10-1844-2_6
44. Wang Z, Ma C, Han H, *et al*. Caregiver burden in Alzheimer's disease: Moderation effects of social support and mediation effects of positive aspects of caregiving. *Int J Geriatr Psychiatry* 2018;33:1198-206. doi:10.1002/gps.4910
45. Parveen S, Morrison V. Predicting caregiver gains: A longitudinal study. *Br J Health Psychol* 2012;17:711–23. doi:10.1111/j.2044-8287.2012.02067.x
46. Kim Y, Schulz R, Carver CS. Benefit finding in the cancer caregiving experience. *Psychosom Medicine* 2007;69:283-91. doi:10.1097/PSY.0b013e3180417cf4
47. Schulz R, Sherwood PR. Physical and mental health effects of family caregiving. *J Soc Work Educ* 2008;44:105-113. doi:10.5175/JSWE.2008.773247702
48. Allen AP, Curran EA, Duggan A, *et al.* A systematic review of the psychobiological burden of informal caregiving for patients with dementia: Focus on cognitive and biological markers of chronic stress. *Neurosci Biobehav Rev* 2017;73:123-64. doi:10.1016/j.neubiorev.2016.12.006
49. Chiao CY, Wu HS, Hsiao CY. Caregiver burden for informal caregivers of patients with dementia: a systematic review. *Int Nurs Rev* 2015; 62:340–50. doi:10.1111/inr.12194
50. Faronbi JO, Faronbi GO, Ayamolowo SJ, *et al*. Caring for the seniors with chronic illness: the lived experience of caregivers of older adults. *Arch Gerontol Geriatr* 2019;82:8–14. doi: 10.1016/j.archger.2019.01.013
51. Geng HM, Chuang DM, Yang F, *et al.* Prevalence and determinants of depression in caregivers of cancer patients: A systematic review and meta-analysis. *Medicine* 2018;97:e11863. doi: 10.1097/MD.0000000000011863

For peer review only - http://bmjopen.bmj.com/site/about/guidelines.xhtml

52. Loh AZ, Tan JS., Zhang MW, *et al*. The global prevalence of anxiety and depressive symptoms among caregivers of stroke survivors. *J Am Med Dir Assoc* 2017;18:111-116. doi:10.1016/j.jamda.2016.08.014
53. Mukherjee A, Biswas A, Roy A, *et al.* Behavioural and psychological symptoms of dementia: correlates and impact on caregiver distress. *Dement Geriatric Cogn Dis Extra* 2017;7:354-365. doi:10.1159/000481568
54. Bell CM, Araki SS, Neumann PJ. The association between caregiver burden and caregiver health-related quality of life in Alzheimer disease. *Alzheimer Dis Assoc Disord* 2001;15:129-36. doi: 10.1097/00002093-200107000-00004
55. Pinquart M, Sorensen S. Differences between caregivers and noncaregivers in psychological health and physical health: A meta-analysis. *Psychol Aging* 2003;18:250-67. doi: 10.1037/0882-7974.18.2.250
56. Vitaliano PP, Zhang J, Scanlan JM. Is caregiving hazardous to one's physical health? A meta-analysis. *Psychol Bull* 2003;129:946-72. doi:10.1037/0033-2909.129.6.946
57. Laudenslager ML."Anatomy of an Illness": Control from a caregiver's perspective. *Brain, Behavior, & Immunity* 2014;36:1–8. doi:10.1016/j.bbi.2013.08.012
58. Koerin BB, Harrigan MP. P.S I love you: Long-distance caregiving. *J Gerontol Soc Work* 2003;40:63-81. doi:10.1300/J083v40n01_05
59. Christie J, Smith GR, Williamson GM, *et al*. Quality of informal care is multidimensional. *Rehabil Psychol* 2009;54:173-81. doi:10.1037/a0015705
60. Smith GR, Williamson GM, Miller LS, *et al*. Depression and quality of informal care: A longitudinal investigation of caregiving stressors. *Psychol Aging* 2011;26:584-91. doi:10.1037/a0022263
61. Hodges LJ, Humphris GM, Macfarlane G. A meta-analytic investigation of the relationship between the psychological distress of cancer patients and their carers. *Soc Sci Med* 2005;60:1-12. doi:10.1016/j.socscimed.2004.04.018
62. Stall NM, Kim SJ, Hardacre KA, *et al*. Association of informal caregiver distress with health outcomes of community-dwelling dementia care recipients: a systematic review. *J Am Geriatr Soc* 2019;67:609-17. doi:10.1111/jgs.15690
63. Kuzuya M, Enoki H, Hasegawa J, *et al*. Impact of caregiver burden on adverse health outcomes in community-dwelling dependent older care recipients. *Am J Geriatr Psychiatry* 2011;19:382-91. doi:10.1097/JGP.0b013e3181e9b98d
64. Doran CM, Kinchin I. A review of the economic impact of mental illness. *Aust Health Rev* 2019;43:43-8. doi:10.1071/AH16115
65. Cooper C, Balamurali TBS, Livingston G. A systematic review of the prevalence and covariates of anxiety in caregivers of people with dementia. *Int Psychogeriatr* 2007;19:175-95. doi:10.1017/S1041610206004297
66. Bledsoe LK, Moore SE, Collins WL. Long distance caregiving: An evaluative review of the literature. *Ageing Int* 2010;*35*:293-310. doi:10.1007/s12126-010-9062-3
67. Benefield LE, Beck C. Reducing the distance in distance-caregiving by technology innovation. *Clin Interv Aging* 2007;2:267-72.
68. Shamseer L, Moher D, Clarke M, *et al*. Preferred reporting items for systematic review and meta-analysis protocols (PRISMA-P) 2015: elaboration and explanation. *BMJ* 2015;349:g7647. doi:10.1136/bmj.g7647
69. Lizarondo L, Stern C, Carrier J, *et al.* Chapter 8: Mixed methods systematic reviews. In: Aromataris E, Munn Z, eds. *Joanna Briggs Institute Reviewer's Manual*. The Joanna Briggs Institute 2017. https://wiki.joannabriggs.org/display/MANUAL/Chapter+8%3A+Mixed+methods+systematic+reviews (accessed 11 Jul 2020)

For peer review only - http://bmjopen.bmj.com/site/about/guidelines.xhtml

70. Shifren, KE. How caregiving affects development: Psychological implications for child, adolescent, and adult caregivers. Washington, DC: American Psychological Association 2009:1-223. doi:10.1037/11849-000
71. Rubira EA, Marcon SR, Belasco AGS, *et al*. Burden and quality of life of caregivers of children and adolescents with chemotherapy treatment for cancer. *Acta Paul Enferm* 2012;25:567-73. doi:10.1590/S0103-21002012005000020
72. Lyonette C, Yardley L. The influence on carer wellbeing of motivations to care for older people and the relationship with the care recipient. *Ageing Soc* 2003;23:487-506. doi:10.1017/S0144686X03001284
73. Abell, N. Assessing willingness to care for persons with AIDS: Validation of a new measure. *Res Soc Work Pract* 2001;11:118-30. doi:10.1177/104973150101100108
74. Radloff LS. The CES-D Scale: A Self-Report Depression Scale for Research in the General Population. *Appl Psychol Meas* 1977;1:385–401. doi:10.1177/014662167700100306
75. Zigmond AS, Snaith RP. The Hospital Anxiety and Depression Scale. *Acta Psychiatr Scand* 1983;67:361–70. doi:10.1111/j.1600-0447.1983.tb09716.x
76. McNair DM, Lorr M, Droppleman LF. Manual for the Profile of Mood States. San Diego, CA: Educational and Industrial Testing Services 1971.
77. Cohen S, Kamarck T, Mermelstein R. A Global Measure of Perceived Stress. *J Health Soc Behav* 1983;24:385–96. doi:10.2307/2136404
78. Zarit SH, Reever KE, Bach-Peterson J. Relatives of the impaired elderly: correlates of feelings of burden. *Gerontologist* 1980;20:649-55. doi:10.1093/geront/20.6.649
79. Cohen S, Hoberman HM. Positive events and social supports as buffers of life change stress. *Journal Appl Soc Psychol* 1983);13:99-125. doi:10.1111/j.1559-1816.1983.tb02325.x
80. Hong QN, Pluye P, Fàbregues S, *et al*. Mixed Methods Appraisal Tool (MMAT), version 2018. Canadian Intellectual Property Office, Industry Canada 2018:1-12.

For peer review only - http://bmjopen.bmj.com/site/about/guidelines.xhtml

Figure Legends

Figure 1

Flow diagram of the planned study selection process adapted from the Preferred Reporting Items for Systematic Reviews and Meta-Analyses (PRISMA) statement [68].

Figure 2

Qualitative synthesis of integrated "qualitised" quantitative data and qualitative data, following the convergent integrated approach as proposed by JBI methodology for mixed-method reviews [69].

For peer review only

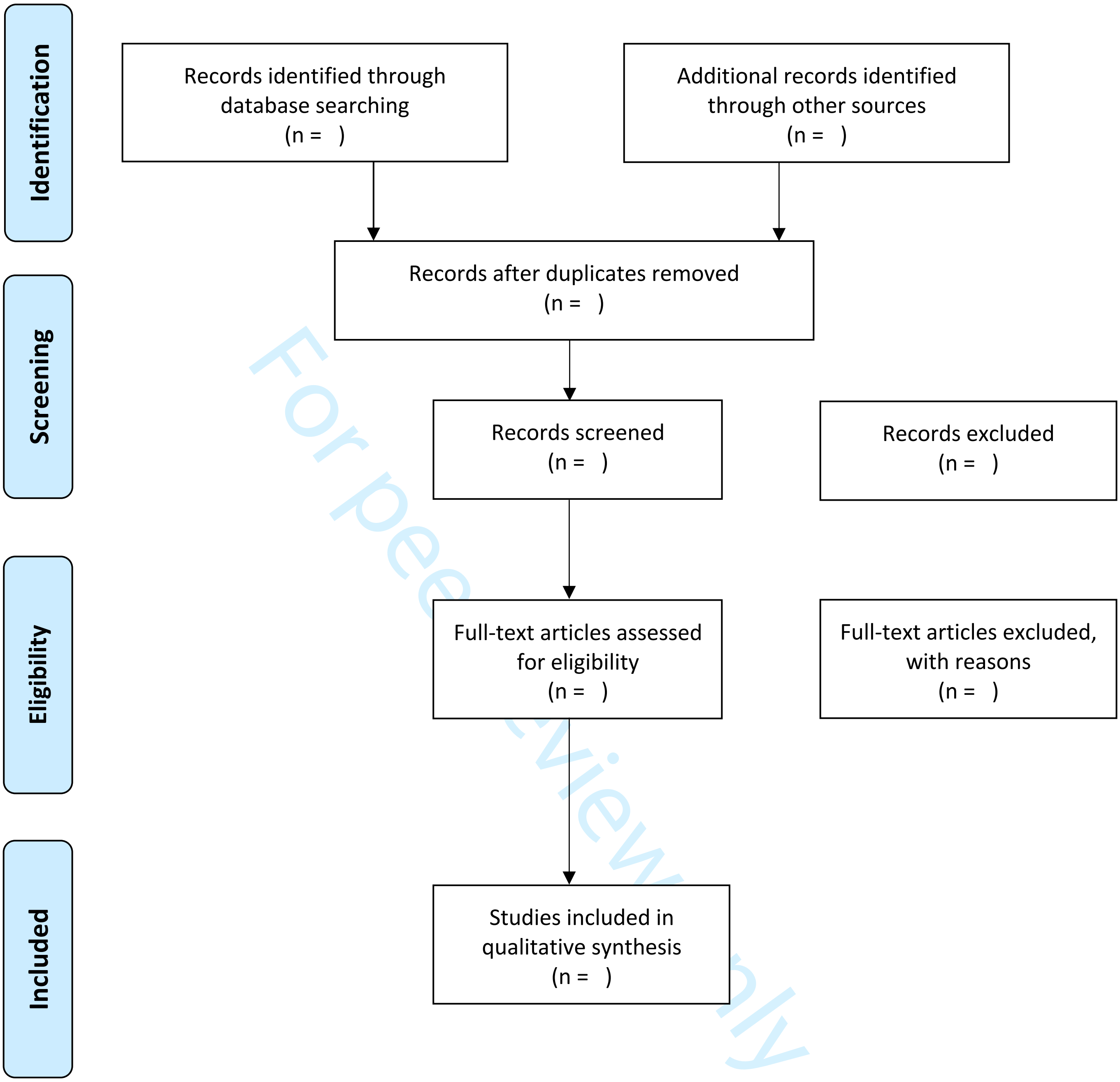

For peer review only

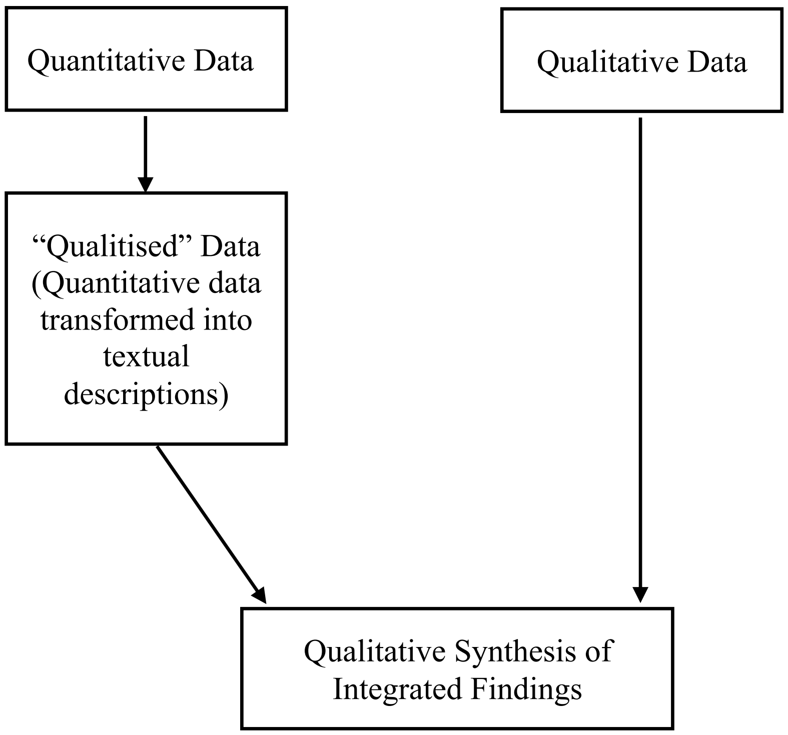
Quantitative Data
Qualitative Data
"Qualitised" Data (Quantitative data transformed into textual descriptions)
Qualitative Synthesis of Integrated Findings

view only

Motivations and willingness to provide care from a geographic distance, and the impact of distance care on caregivers' mental and physical health: A mixed-method systematic review protocol

PRISMA-P (Preferred Reporting Items for Systematic review and Meta-Analysis Protocols) 2015 checklist: recommended items to address in a systematic review protocol*

Section and topic	Item No	Checklist item	Location in text (page number)
**ADMINISTRATIVE INFORMATION**			
Title:			
Identification	1a	Identify the report as a protocol of a systematic review	1
Update	1b	If the protocol is for an update of a previous systematic review, identify as such	N/A
Registration	2	If registered, provide the name of the registry (such as PROSPERO) and registration number	8
Authors:			
Contact	3a	Provide name, institutional affiliation, e-mail address of all protocol authors; provide physical mailing address of corresponding author	1
Contributions	3b	Describe contributions of protocol authors and identify the guarantor of the review	13
Amendments	4	If the protocol represents an amendment of a previously completed or published protocol, identify as such and list changes; otherwise, state plan for documenting important protocol amendments	8
Support:			
Sources	5a	Indicate sources of financial or other support for the review	13
Sponsor	5b	Provide name for the review funder and/or sponsor	13
Role of sponsor or funder	5c	Describe roles of funder(s), sponsor(s), and/or institution(s), if any, in developing the protocol	13
**INTRODUCTION**			
Rationale	6	Describe the rationale for the review in the context of what is already known	3-7
Objectives	7	Provide an explicit statement of the question(s) the review will address with reference to participants, interventions, comparators, and outcomes (PICO)	7
**METHODS**			
Eligibility criteria	8	Specify the study characteristics (such as PICO, study design, setting, time frame) and report characteristics (such	8-10

For peer review only

		as years considered, language, publication status) to be used as criteria for eligibility for the review	
Information sources	9	Describe all intended information sources (such as electronic databases, contact with study authors, trial registers or other grey literature sources) with planned dates of coverage	10
Search strategy	10	Present draft of search strategy to be used for at least one electronic database, including planned limits, such that it could be repeated	10 & Supplementary file 2
Study records:			
Data management	11a	Describe the mechanism(s) that will be used to manage records and data throughout the review	10-11
Selection process	11b	State the process that will be used for selecting studies (such as two independent reviewers) through each phase of the review (that is, screening, eligibility and inclusion in meta-analysis)	10-11
Data collection process	11c	Describe planned method of extracting data from reports (such as piloting forms, done independently, in duplicate), any processes for obtaining and confirming data from investigators	11
Data items	12	List and define all variables for which data will be sought (such as PICO items, funding sources), any pre-planned data assumptions and simplifications	11 & Supplementary file 3
Outcomes and prioritization	13	List and define all outcomes for which data will be sought, including prioritization of main and additional outcomes, with rationale	9-10
Risk of bias in individual studies	14	Describe anticipated methods for assessing risk of bias of individual studies, including whether this will be done at the outcome or study level, or both; state how this information will be used in data synthesis	11
Data synthesis	15a	Describe criteria under which study data will be quantitatively synthesised	N/A
	15b	If data are appropriate for quantitative synthesis, describe planned summary measures, methods of handling data and methods of combining data from studies, including any planned exploration of consistency (such as I^2, Kendall's τ)	N/A
	15c	Describe any proposed additional analyses (such as sensitivity or subgroup analyses, meta-regression)	N/A
	15d	If quantitative synthesis is not appropriate, describe the type of summary planned	12 & Figure 2
Meta-bias(es)	16	Specify any planned assessment of meta-bias(es) (such as publication bias across studies, selective reporting within studies)	10-11
Confidence in cumulative evidence	17	Describe how the strength of the body of evidence will be assessed (such as GRADE)	N/A

N/A= Not Applicable

*** It is strongly recommended that this checklist be read in conjunction with the PRISMA-P Explanation and Elaboration (cite when available) for important clarification on the items. Amendments to a review protocol should be tracked and dated.** **The copyright for PRISMA-P (including checklist) is held by the PRISMA-P Group and is distributed under a Creative Commons Attribution Licence 4.0.**

From: Shamseer L, Moher D, Clarke M, Ghersi D, Liberati A, Petticrew M, Shekelle P, Stewart L, PRISMA-P Group. Preferred reporting items for systematic review and meta-analysis protocols (PRISMA-P) 2015: elaboration and explanation. BMJ. 2015 Jan 2;349(jan02 1):g7647.

For peer review only

Motivations and willingness to provide care from a geographic distance, and the impact of distance care on caregivers' mental and physical health: A mixed-method systematic review protocol

Supplementary File 2: Search Strategy – PubMed

#	Search Terms
#1	Distance caregiving [Title/Abstract] OR<br>Distance caregiver* [Title/Abstract] OR<br>Distant caregiving [Title/Abstract] OR<br>Distant caregiver* [Title/Abstract] OR<br>Distance carer* [Title/Abstract] OR<br>Distant carer* [Title/Abstract] OR<br>Spatial proximity + care [Title/Abstract] OR<br>Care from afar [Title/Abstract]] OR<br>Caregiving from afar [Title/Abstract] OR<br>Caregiver* from afar [Title/Abstract] OR<br>Proximate caregiver* [Title/Abstract] OR<br>Proximate caregiving [Title/Abstract] OR<br>Proximate carer* [Title/Abstract] OR<br>Out of town caregiver* [Title/Abstract] OR<br>Immigrant caregiver* [Title/Abstract] OR<br>Transnational caregiver* [Title/Abstract] OR<br>Transnational caregiving [Title/Abstract]

For peer review only

**#2**	motiv* [Title/Abstract] OR<br><br>motivation* to care [Title/Abstract] OR<br><br>motivation* to provide care [Title/Abstract] OR<br><br>oblig* [Title/Abstract] OR<br><br>filial [Title/Abstract] OR<br><br>duty [Title/Abstract] OR<br><br>guilt* [Title/Abstract] OR<br><br>reciproc* [Title/Abstract] OR<br><br>love [Title/Abstract] OR<br><br>willing* [Title/Abstract] OR<br><br>willingness to care [Title/Abstract] OR<br><br>willingness to provide care [Title/Abstract] OR<br><br>love [MeSH Terms]
**#3**	depress* [Title/Abstract] OR<br><br>distress [Title/Abstract] OR<br><br>stress [Title/Abstract] OR<br><br>mood [Title/Abstract] OR<br><br>anxiety [Title/Abstract] OR<br><br>well being [[Title/Abstract] OR<br><br>mental health [Title/Abstract] OR<br><br>caregiver burden [Title/Abstract] OR<br><br>burden [Title/Abstract] OR<br><br>physical symptoms [[Title/Abstract] OR<br><br>depression [MeSH terms] OR<br><br>anxiety [MeSH terms] OR<br><br>anxiety disorders [MeSH] OR<br><br>mood disorders [MeSH terms] OR

For peer review only

	mental health [MeSH terms] OR<br><br>health [MeSH terms]
**#4**	OR #2 / #3
**#5**	#1 AND #4

Note. * = truncation symbol to identify variations and plurals of words

For peer review only

Motivations and willingness to provide care from a geographic distance, and the impact of distance care on caregivers' mental and physical health: A mixed-method systematic review protocol

Supplementary File 3: Data extraction form (Adapted from Lizarondo *et al.*) [69]

**Study Identification Features**	
**Reviewer**	
**Article Author(s)**	
**Study Title**	
**Date of Publication**	
**Publication Type**	• Research Article<br>- Journal Name:<br>- Record Number:<br>• PhD Thesis/Dissertation<br>• Conference Paper<br>• Other Please Specify: ____________________
**Country of Study**	
**Research Aims and Objectives**	
**Methodology**	
**Type of Study (Quantitative, Qualitative or Mixed-method study)**	
**Methodology/Study Design (e.g. Cross-sectional study, Phenomenology)**	
**Method of Analysis (e.g. Thematic analysis, Hierarchical regression analyses)**	
**Method(s) of Data Collection (e.g. Online survey, Interviews, Focus groups)**	
**Inclusion/Exclusion Criteria**	
**Final Sample Size**	
**Recruitment Rate**	
**Drop-out Rate**	
**Time Points (If applicable)**	

For peer review only

**Drop-out Rate for Follow-up Points (If applicable)**		
**Participants' Characteristics**		
**Age**		*$\bar{x}$ (SD)*
**Gender**		*%N*
**Ethnicity**		*%N*
**Care Recipient's Diagnosis**		
**Relationship to Care Recipient**		
**Time Caring**		
**Other Care Experience**		
**Employment Status**		
**Phenomena of Interest**		
**Concepts (C1) - Motivations to Care and/or Willingness to Care**	• Motivations to Care from a Geographic Distance<br>• Willingness to Care from a Geographic Distance<br>• Willingness to Perform Specific Care Tasks (e.g. emotional, nursing or instrumental care tasks)	
**Concepts (C2) – Mental and Physical Health Outcomes**	• Depression<br>• Anxiety<br>• Emotional Distress<br>• Caregiver Burden<br>• Perceived Physical Health<br>• Physical Symptoms	
**Context-related Information**		
**Geographic Distance from the Care Recipient (e.g. assessed using miles or travel time)**		
**Quantitative Findings**		
**Measures**		
**Key Findings**		
**Qualitative Findings**		
**Identified Themes or Subthemes**		
**Author's Conclusions**		
**Reviewer's Comments**		
